# A novel family of integrases associated with prophages and genomic islands integrated within the tRNA-dihydrouridine synthase A (*dusA*) gene

**DOI:** 10.1093/nar/gkv337

**Published:** 2015-04-16

**Authors:** Daniel N. Farrugia, Liam D. H. Elbourne, Bridget C. Mabbutt, Ian T. Paulsen

**Affiliations:** Department of Chemistry and Biomolecular Sciences, Macquarie University, Sydney, NSW 2109, Australia

## Abstract

Genomic islands play a key role in prokaryotic genome plasticity. Genomic islands integrate into chromosomal loci such as transfer RNA genes and protein coding genes, whilst retaining various cargo genes that potentially bestow novel functions on the host organism. A gene encoding a putative integrase was identified at a single site within the 5′ end of the *dusA* gene in the genomes of over 200 bacteria. This integrase was discovered to be a component of numerous genomic islands, which appear to share a target site within the *dusA* gene. *dusA* encodes the tRNA-dihydrouridine synthase A enzyme, which catalyses the post-transcriptional reduction of uridine to dihydrouridine in tRNA. Genomic islands encoding homologous *dusA*-associated integrases were found at a much lower frequency within the related *dusB* and *dusC* genes, and non-*dus* genes. Excision of these *dusA*-associated islands from the chromosome as circularized intermediates was confirmed by polymerase chain reaction. Analysis of the *dusA*-associated islands indicated that they were highly diverse, with the integrase gene representing the only universal common feature.

## INTRODUCTION

Since the discovery that the genomes of *Escherichia coli* K12 and O157:H7 differed by more than a megabase of DNA (1), regions of genomic variability have consistently been observed in the genomes of prokaryotic organisms. The acquisition of genes through lateral gene transfer is undoubtedly one of the major drivers of this variability. These variable regions are currently classified by three overlapping terminologies. Mobile genetic elements (MGEs) are a collective of well-characterized genetic entities that are capable of intracellular or intercellular translocation, such as prophages and insertion sequences (2). Genomic islands (GEIs) are discrete gene clusters containing features suggestive of lateral gene transfer, such as atypical nucleotide content and mobilization genes, and include some MGE classes (3,4). Regions of genomic plasticity (RGPs) are genomic regions that are variable compared to related organisms, without any assumption regarding their evolutionary or genetic basis, which include both laterally acquired regions and plasticity arising through other recombinative mechanisms (5,6).

GEIs carry multiple genes that may encode advantageous traits, including antimicrobial resistance, virulence and metabolic pathways (7). They also frequently encode a high number of hypothetical proteins of unknown function, suggesting that GEIs act as reservoirs of genetic novelty (8). GEIs are classically inserted within a narrow assortment of transfer RNA and transfer-messenger RNA genes (9–11), but are also known to integrate into various protein coding genes (6,12). GEIs also typically possess an integrase/recombinase gene, facilitating chromosomal integration and excision (2). Recombinases fall into two classical protein superfamilies, known as the tyrosine and serine recombinases, characterized by the namesake nucleophilic amino acid residue that catalyses DNA cleavage and ligation (13).

Prophages, viral genomes integrated within the chromosomes of host bacterial, are MGEs often classified as GEIs, and possess most of the aforementioned features of GEIs (3). However, an overwhelming majority of these elements encode tyrosine recombinases, the function of which is often limited to prophage lysogeny (2). An exception is occasional presence of phage morons, genes acquired from the host chromosome that are uninvolved in phage function, capable of autonomous transcription (14).

The accelerating release of bacterial genome sequences has fuelled the discovery of novel GEI variants. However, pathogens constitute the bulk of available bacterial genome sequences, particularly those of the Proteobacterial lineage, which is reflective of the GEIs and MGEs that are discovered (2,15). Some of the most prominent GEI families are resistance or pathogenicity islands, limited to a single genus or species, such as the *Acinetobacter baumannii* antibiotic resistance island (Tn*AbaR*) (16), *Salmonella* genomic island 1 (SGI1) (17), *Vibrio cholera* SXT (18) and the *Yersinia* high-pathogenicity island (19).

Analysis conducted on several *Acinetobacter baumannii* and *Pseudomonas* sp. genomes, previously sequenced by our research group (20–23), revealed the presence of a gene encoding a putative integrase adjacent to the 5′ end of the *dusA* gene. *dusA* encodes the tRNA-dihydrouridine synthase A enzyme and is a member of the dihydrouridine synthase (DUS) family, an enzyme family present in many prokaryotes and eukaryotes (24), that functions in the post-transcriptional reduction of uridine in the D-loop of tRNA to 5,6-dihydrouridine (25).

In this manuscript, we outline a novel family of integrases present in disparate GEIs and prophages, typically inserted within the *dusA* gene in over 200 sequenced Proteobacterial organisms. GEIs encoding these *dusA*-associated integrases (DAIs) consisted of a highly mosaic cargo gene pool, encoding various functions including bacteriophage lysogeny, heavy metal resistance, metabolic augmentation and conjugative transfer, in addition to numerous uncharacterized hypothetical proteins. Excision of *dusA*-associated GEIs and prophages were demonstrated through polymerase chain reaction (PCR) amplification of both the circularized intermediate and the remnant chromosomal junction. Sequencing of these amplicons, and subsequent bioinformatic analyses, identified a conserved sequence motif likely involved in the targeting and integration of these GEIs into the *dusA* gene, as well as some non-congnate target genes.

## MATERIALS AND METHODS

### Bioinformatic parameters and analyses

BLAST (26) searches to identify integrase homologues were conducted using the default parameters and the NCBI ‘non-redundant protein sequences’ (nr) database, except the maximum target sequences were set to 20 000 and an e-value cutoff of 1e-20 was used. Integrases that were either below this cutoff, or belonging to non-redundant records lacking genome sequences, were excluded from this analysis. Integrases were considered *dusA*-associated if the 5′ end of the integrase gene adjoined the 5′ end of the *dusA* gene (Figure 3). However, integrases found not to be *dusA*-associated, but greater than the 1e-20 cutoff, were included in this analysis. Multiple alignments of protein and nucleotide sequences were carried out using ClustalW (27) and ClustalOmega (28). Neighbour-joining phylogenetic analysis and protein identity/similarity matrices were generated from multiple alignments using MEGA5 (29) and MatGAT (30), respectively. TreeGraph2 (31) and UGENE (32) were routinely used for phylogenetic tree annotation and genome browsing, respectively. Logo consensus sequences were generated using WebLogo (33). The gene content of numerous *dusA*-associated GEIs (Supplementary Table S1) were compared reciprocally by BLASTP+ (34) to identify putative orthologues, using an e-value cutoff of 1e-5. Oligonucleotides used in PCR (Supplementary Table S2) were designed with the aid of Primer3 (35) with the oligonucleotide length, melting temperature and GC% parameters set to 20–25 nt, 55–60°C and 30–50%, respectively.

**Figure 1.**
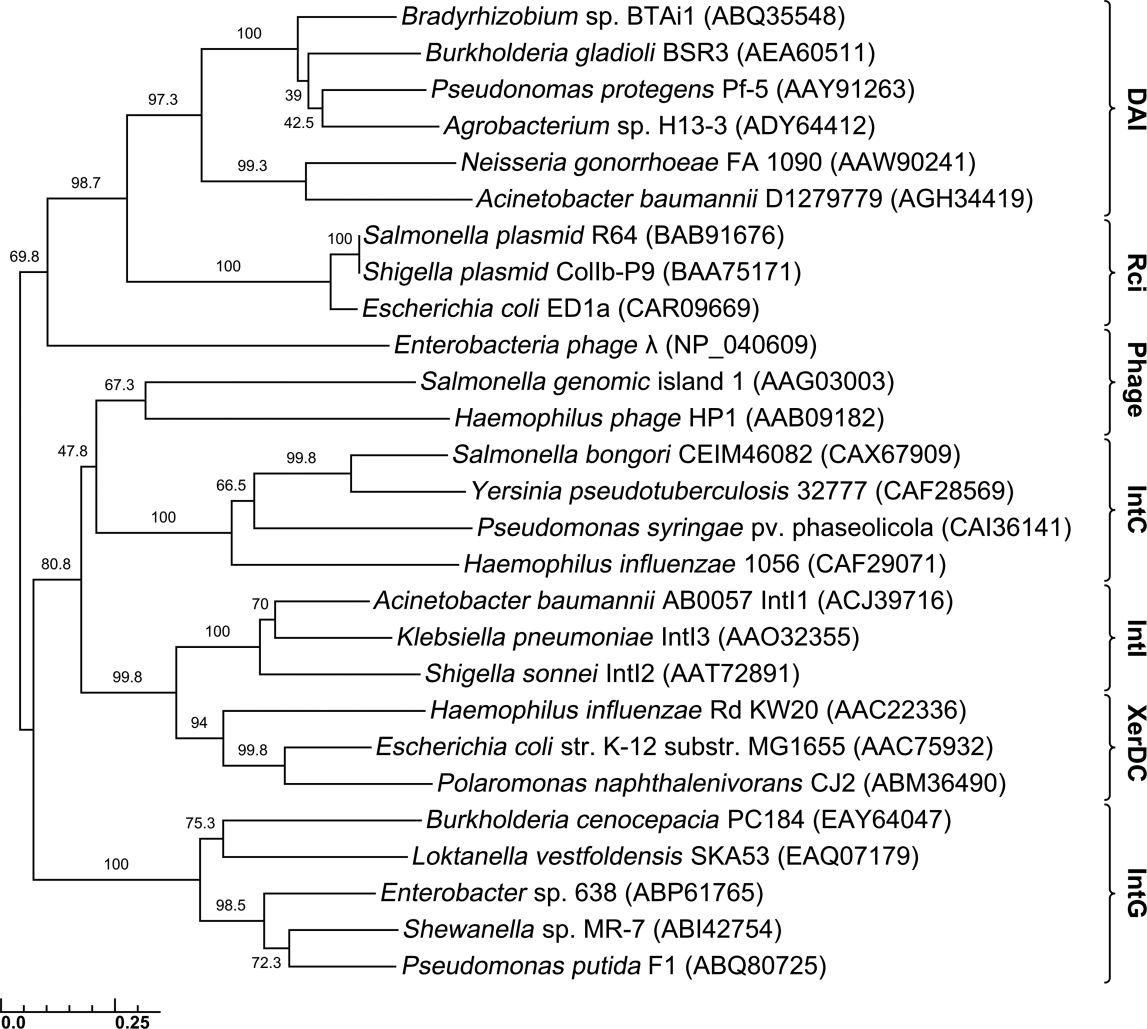
The inferred phylogenetic relationship of the DAIs in relation to representative tyrosine recombinases of the shufflon-specific DNA recombinase (Rci), phage integrase (Phage), integrative and conjugative element (IntC), integron integrase (IntI), site-specific recombinase (XerDC) and genomic island integrase (IntG) families. Protein accession numbers of sequences used to generate this phylogenetic tree are in brackets. The interior values are the bootstrap probabilities after 1000 replicates.

**Figure 2.**
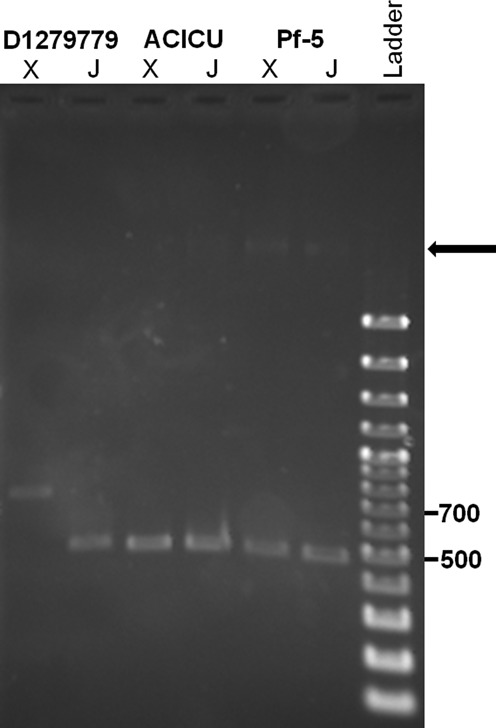
PCR detection of excised *dus*-associated GEIs (X) and restored *dusA*/*dusB* chromosomal junctions (J) in *Acinetobacter baumannii* D1279779 (740 bp and 494 bp), *A. baumannii* ACICU (534 bp each) and *Pseudomonas protegens* Pf-5 (472 bp and 485 bp). The faint high molecular weight bands originate from genomic DNA template added to the PCR reactions for *A. baumannii* ACICU and *P. protegens* Pf-5 (black arrow). Covalently closed circular DNA (cccDNA) was used as template for *A. baumannii* D1279779.

**Figure 3.**
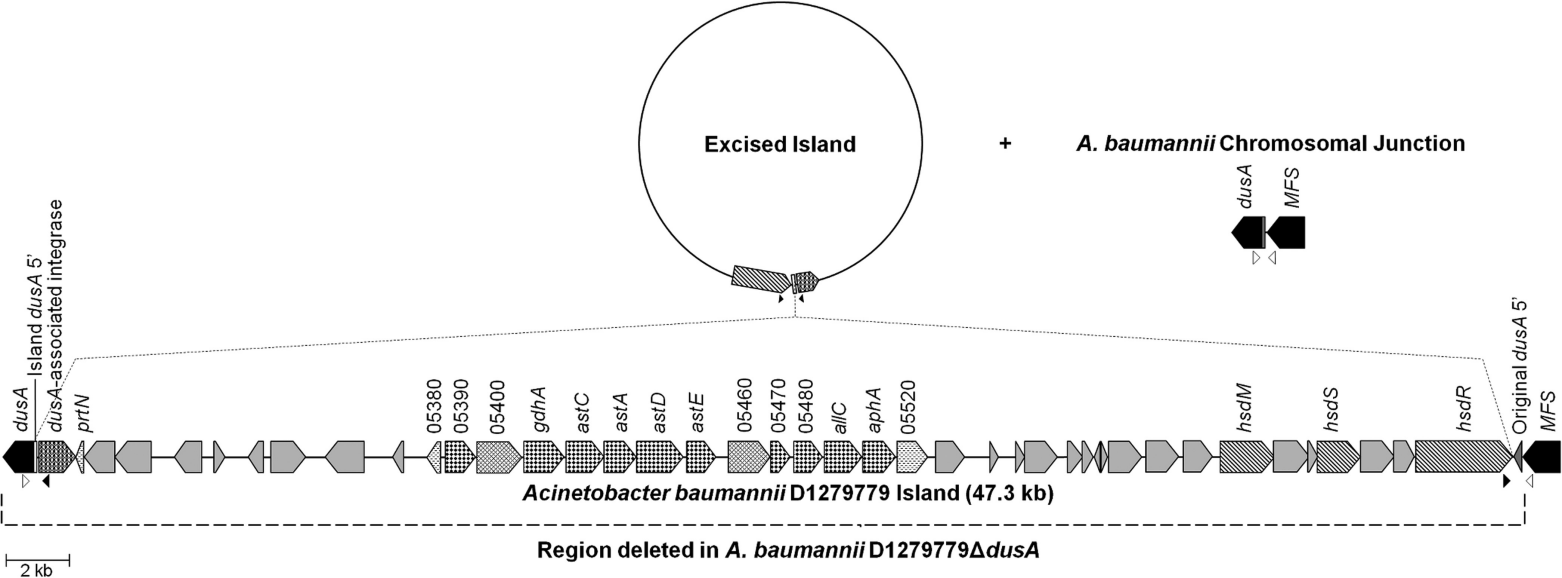
Excision and integration of the *Acinetobacter baumannii* D1279779 *dusA*-specific GEI. The *dusA*-specific GEIs consist of a DAI (plaid) that putatively catalyses excision of the GEI as a circular intermediate, as well as its integration into the 5′ end of the chromosomal tRNA-dihydrouridine synthase A (*dusA*) gene (black). The 5′ portion of the *dusA* gene affected by the integration is replaced by a new 5′ end (white) provided by the GEI, with the original *dusA* 5′ end (dark grey) forming the outer boundary of the island. This particular GEI variant encodes several hypothetical proteins (light grey) as well as proteins putatively involved in metabolism (diamonds), transport (outlined diamonds), transcriptional regulation (dashed lines), and type I restriction-modification (diagonal lines). As is true of other *A. baumannii* strains, the *dusA*-associated GEIs are flanked by a gene encoding a putative major facilitator superfamily transporter (*MFS*) (black). The binding sites of oligonucleotides used to detect excision of the GEI (black triangles) and restoration of the *dusA* sequence (white triangles) are indicated. This figure is drawn to scale with the exception of the excised island, which is displayed at 60% scale.

### Bacterial strains, culture conditions and genomic DNA extraction

*Acinetobacter baumannii* strains D1279779 and ACICU, and *Pseudomonas protegens* Pf-5 were cultured overnight in Müeller-Hinton broth (MH) (Oxoid) at 37°C and 25°C, respectively. Broth microdilution minimal inhibitory concentration (MIC) testing was conducted as described previously (36), to determine the resistance level of these organisms against the antibiotic mitomycin C (MMC) (AG Scientific), with a minimum of three temporally distinct replicates. The MMC MICs for *A. baumannii* strains D1279779 and ACICU were determined to be 50 μg/ml, whilst the MIC for *P. protegens* Pf-5 was 0.8 μg/ml. Induction of *dusA/dusB*-associated GEI excision was modified from a previous protocol for excision of integrative elements (37) as follows: 5 ml of mid-exponential (OD_600nm_ 0.6) *A. baumannii* and *P. protegens* subcultures (in MH) were exposed to subinhibitory concentrations of MMC. *A. baumannii* ACICU and *P. protegens* Pf-5 were treated with 0.5x MIC MMC for up to 2 h, whilst *A. baumannii* D1279779 was treated with 0.75x MIC MMC for 1 h. Total genomic DNA from ACICU and Pf-5 was isolated using the PureLink Genomic DNA Mini Kit (Invitrogen), whilst total covalently closed circular DNA (cccDNA) from *A. baumannii* D1279779 was isolated using the Wizard® Plus SV Minipreps DNA Purification System (Promega), as per the manufacturers’ protocols.

### PCR detection of GEI excision

PCR amplicons confirming the excision of *dusA*-associated GEIs and restoration of the wild-type *dusA* (and *dusB*) sequence (Figure 3) were generated with Platinum *Taq* DNA Polymerase High Fidelity (Invitrogen) at 35 cycles, using the primers listed in Supplementary Table S2 and 100 ng of total genomic DNA, or 50 ng of total cccDNA in the case of *A. baumannii* D1279779. The amplicons were purified using the Wizard^®^ SV Gel and PCR Clean-Up System (Promega) as per the manufacturer's instructions, and were sequenced bidirectionally using BigDye Terminator v3.1 chemistry dideoxysequencing (Applied Biosciences). All sequencing reactions and purifications were conducted at the Australian Genome Research Facility (Westmead, NSW, Australia). The resultant chromatograms were edited and assembled in ChromasPro (Technelysium Pty. Ltd.) and crosschecked against their respective genome sequences.

### Deletion of D1279779_RGP05 and *in trans dusA* complementation

The strains and primers used in the following procedures are listed in Table 1 and Supplementary Table S2, respectively. Deletion of a 48.3 kb region extending from the original *dusA* 5′ end to the *dusA* 3′ end of *A. baumannii* D1279779 (Figure 3) was deleted as per a previous method (38) with several alterations. Mating of *A. baumannii* D1279779 and *E. coli* S17-1 (39) harbouring pJQFRT_05270 was conducted on LB agar at 37°C for 7 h. Cells were collected and resuspended in 1 ml of 10 mM MgSO_4_, serially diluted, plated onto CHROMagar *Acinetobacter* (Dutec Diagnostics) containing 10 μg/ml gentamycin and incubated overnight at 37°C. In brief, CHROMagar *Acinetobacter* is a proprietary chromogenic agar that is selective for *Acinetobacter* spp. organisms, which appear as bright red colonies, whilst other organisms are mostly inhibited; *E. coli* S17-1 uninhibited by the media appear as metallic blue colonies. D1279779 colonies resistant to gentamycin were screened for integration of pJQFRT_05270 using the primers P1_RGP05_FOR and FRT-SP6R. The D1279779 transformant was subsequently mated with *E. coli* S17-1 harbouring pKFRT/FLP_05691 as above, except transformants were selected on CHROMagar *Acinetobacter* containing 15 μg/ml gentamycin and 30 μg/ml kanamycin. D1279779 colonies resistant to both gentamycin and kanamycin were screened for pKFRT/FLP_05691 integration using the primers FRT-leftF and P4_RGP05_REV. Excision of the DNA sandwiched between the two plasmids and screening of desired deletions were conducted as per the original method (38), except all incubation steps were conducted with LB or LB agar at 37°C. *In trans* complementation of the resultant Δ*dusA* mutant was accomplished using the *E. coli*/*A. calcoaceticus* shuttle vector pWH1266 (40), containing *dusA* and its native promoter originating from either *A. baumannii* D1279779 (DAI present) or *A. baumannii* ATCC 17978 (41) (DAI absent), cloned into the BamHI site. The resultant constructs were electroporated into *A. baumannii* D1279779Δ*dusA* and selected on LB agar plates containing 50 μg/ml carbenicillin.

**Table 1.** Bacterial strains and plasmids utilized in this study with relevant genotypes indicated

Bacterial strains	Description and relevant genotype	Reference
*Acinetobacter baumannii* ACICU	Intact *dusA*, *dusB*::pA_ICU_30^a^	(43)
*Acinetobacter baumannii* ATCC 17978	Intact *dusA*	(41)
*Acinetobacter baumannii* D1279779	Wild-type strain, *dusA*::D1279779_RGP05	(23)
*A. baumannii* D1279779Δ*dusA*	ΔABD1_05270-05691^b^	This study
*A. baumannii* D1279779Δ*dusA* (Empty vector)	D1279779Δ*dusA* + pWH1266	This study
*A. baumannii* D1279779Δ*dusA* (Island)	D1279779Δ*dusA* + pWH5270	This study
*A. baumannii* D1279779Δ*dusA* (Wild-type)	D1279779Δ*dusA* + pWH0562	This study
*Pseudomonas protegens* Pf-5	*dusA*::Prophage 03	(20,45)
*Escherichia coli* S17-1	Donor strain for conjugation	(39)
**Plasmids**		
pJQFRT	Gene replacement vector, FRT, *sacB*, Gm^R^	(38)
pKFRT/FLP	Gene replacement vector, FRT, *flp*, *tetR*, Km^R^	(38)
pJQFRT_05270	1 kbp region, upstream of *dusA*, cloned into BamHI and SacI pJQFRT sites	This study
pKFRT/FLP_05691	1 kbp region, downstream of *dusA* 5′, cloned into BamHI and BspEI pJQFRT sites	This study
pWH1266	Ap^R^, Tc^R^	(40)
pWH5270	*A. baumannii* D1279779 *dusA* and native promoter cloned into pWH1266 BamHI site in *tet* gene, Ap^R^	This study
pWH0562	*A. baumannii* ATCC 17978 *dusA* and native promoter cloned into pWH1266 BamHI site in *tet* gene, Ap^R^	This study

^a^The *dusA/B*::IslandX genotypes denote disruption of the *dusA*/*dusB* nucleotide sequence by the insertion of the *dus*-associated islands.

^b^Deletion of a 48.3 kb region consisting of *dusA*, D1279779_RGP05 and the original *dusA* 5′ end.

Ap: ampicillin; Gm: gentamycin; Km: kanamycin; Tc: tetracycline.

### Phenotype microarray testing

Biolog phenotype microarray (PM) assays of the five isogenic *A. baumannii* D1279779 strains were conducted as previously (23), except where antibiotic selection was required for propagation of pWH1266 and its variants on agar. PMs are conducted within a series of 96-well plates containing various lyophilized compounds, and response through respiration is measured using a tetrazolium dye indicator (42). Plates PM1-2 were used to test carbon utilization, and plates PM9-10 were used to test responses to osmotic, ionic and pH stressors.

## RESULTS AND DISCUSSION

### Identification of numerous GEIs associated with the *dusA* gene

In the course of bioinformatic analysis conducted on *Acinetobacter baumannii* and *Pseudomonas* sp. genomes previously sequenced by our research group (20–23), we serendipitously observed that these two groups of organisms occasionally encoded a homologous integrase gene associated with the 5′ end of the *dusA* gene. Alterations in the 5′ sequence of *dusA* were evident in organisms that encoded this integrase, compared to related strains lacking it.

This initial analysis led to the subsequent discovery of other DAI homologues in previously predicted GEIs of *Acinetobacter* and *Pseudomonas* that were not identified as having integrated into *dusA*. Such elements included D1279779_RGP05 of *A. baumannii* D1279779 (23), pA_ICU_30 of *A. baumannii* ACICU (*dusB*) (43), G08 of *A. baumannii* AB0057 and AYE (44), RGP56 of *P. aeruginosa* PA7 (21) and Prophage 03 of *P. protegens* Pf-5 (45). A handful of other GEIs in published genomes were associated with the *dusA* gene in *Escherichia coli* (46,47), *Bradyrhizobium* sp. BTAi1 (48) and *Vibrio parahaemolyticus* AQ3354 (49). However, none of these reports elucidated that these islands encoded a homologous integrase with a preference for a common insertion site.

Further BLAST analysis uncovered the presence of numerous other DAI homologues in over 200 bacterial genomes, typically located near the 5′ end of the *dusA* gene. In each case, the DAI-encoding gene was flanked at the 3′ end with a gene cluster that had typical hallmarks of laterally acquired DNA, i.e. they were not conserved in the core genome of related strains and they possessed atypical trinucleotide content. These genomic regions appeared to vary greatly in gene content and estimated size, typically in the range of 30–60 kb. Taken together, this data suggested that there may be a number of GEIs encoding a distinct integrase with an affinity for the *dusA* gene.

### The DAIs are novel members of the tyrosine recombinase superfamily

BLASTP-based analysis suggested that the DAIs were uncharacterized members of the tyrosine recombinase superfamily, and their closest characterized relative was the shufflon-specific DNA recombinase (Rci) family. Rci is encoded in incompatibility group I Enterobacteriaceal plasmids, such as R64 (50) and R621a (51), and mediate the site-specific recombination of the mobile shufflon region located at the C-terminus of the *pilV* gene, determining recipient specificity in liquid mating (52). An alignment of protein sequences from representative tyrosine recombinases against several DAIs confirmed that the latter are members of the tyrosine recombinase superfamily, as they contain the RHRH tetrad, and nucleophilic tyrosine, characteristic of this protein superfamily (53,54) (Supplementary Figure S1). Phylogenetic analysis derived from the alignment of these tyrosine recombinases confirmed that the closest relative to the DAI family is the Rci family. Additionally, this analysis established that the DAI family forms the basis of a novel tyrosine recombinase family, as they are phylogenetically distinct from the integrases of bacteriophages, integrons, integrative conjugative elements and other GEIs (Figure 1).

Additional sequence alignments conducted with representative DAI and Rci recombinase proteins revealed, in addition to the conserved RHRH/Y pentad, another 15 amino acid residues that were conserved between the two protein families (Supplementary Figure S2). This alignment also demonstrated that Rci has an extended C-terminus relative to the DAIs (Supplementary Figure S2), previously established to be essential in the recombination of asymmetrical recombination sites in the shufflon (55). Further mutagenesis of the conserved RHR/Y residues in Rci had confirmed their essentiality for recombination activity, but not DNA binding (56). Both phylogenetic analysis and sequence alignments suggested that whilst the DAIs and Rci are related, the function of these two tyrosine recombinases may be divergent.

### The DAIs are exclusive to three of seven Proteobacterial lineages

The DAI protein sequence from *A. baumannii* D1279779 (protein accession: AGH34419) was utilized in BLASTP searches to retrieve other DAIs from publicly available genome sequences. A total of 258 unique integrase variants (including that of D1279779) were retrieved, with 213 (82.6%) of these found to be associated with *dusA*. The other 45 integrases were either associated with an unknown gene, due to incomplete genome sequences (7 hits), or were associated with genes other than *dusA* (32 hits), including those encoding a tRNA^Arg^ (*Pseudogulbenkiania ferrooxidans* 2002), a glutamate-ammonia ligase (*Rhodobacter sphaeroides* ATCC 17029), a sugar transporter (*Enterobacter radicincitans* DSM 16656), an aminobenzoyl-glutamate transporter (*abgT*) (*Klebsiella pneumonia*) and the related *dusB* and *dusC* genes (16 hits). Another six hits originated from bacteriophage genomes of *Burkholderia* phages AH2, BcepMigl, BcepIL02, and DC1, Enterobacteria phage mEp460 and *Pseudomonas* phage H66.

There were several instances of organisms harbouring more than one DAI in their genome (Supplementary Figure S3). For instance, *Acinetobacter* sp. ANC 3789 harboured three integrase orthologues associated with the *dusA*, *dusB* and *rluA* genes, the latter of which also encodes an enzyme involved in tRNA-modification, tRNA-pseudouridylate synthase. In other instances, the genome of *Alteromonas macleodii* str. ‘Deep ecotype’ encoded two copies of the *dusA* gene along with two identical *dusA*-associated GEIs, whilst *Simonsiella muelleri* ATCC 29453 encoded a pair of *dusB* and *dusC*-associated integrases.

The amino acid identities of the 213 DAIs (adjacent to *dusA*), relative to that of *A. baumannii* D1279779, ranged from 22.8% (*Pseudogulbenkiania* sp. NH8B) to 97.6% (*Acinetobacter baumannii* OFIC143), and were identified exclusively within organisms of the Alphaproteobacteria, Betaproteobacteria and Gammaproteobacteria lineages. No DAI homologues were identified in Deltaproteobacterial or Epsilonproteobacterial organisms, which may be linked to the absence of *dusA* in these lineages. BLASTP searches against these taxonomic classes confirmed the complete absence of *dusA* from sequenced representatives of the Epsilonproteobacteria (24), and revealed that *dusA* is encoded within some sequenced Myxococcales of the Deltaproteobacteria class. Organisms within the recently proposed Zetaproteobacteria class (57) do not encode *dusA*, whilst a more recently proposed class, Acidithiobacillia (58), does encode *dusA*, though currently available genome sequences do not encode a DAI. An exception is genome of *Thermithiobacillus tepidarius* DSM 3134, which instead of *dusA* encodes *dusB*. The *dusA* gene is also known to exist in some non-Proteobacterial organisms including Cyanobacteria and Viridiplantae (24).

The phylogeny of the DAI protein family was inferred with a neighbour-joining analysis based on a multiple alignment of the integrase protein sequences. It was found that majority of these integrases had clustered according to their genus level relationships, particularly in the overrepresented Gammaproteobacterial genera *Acinetobacter*, *Escherichia* and *Pseudomonas* (Supplementary Figure S3). The integrases of *Acinetobacter* and *Neisseria* appear to be collaterally related, in that each genus is part of a distinct taxonomic class, yet the phylogeny suggests a common ancestral integrase (Supplementary Figure S3). Integrases that were not associated with *dusA* were generally disparate from other integrases in closely related organisms (Supplementary Figure S3), particularly in the case of the *Acinetobacter dusB*-associated integrases, such as the integrase present in the pA_ICU_30 island of *A. baumannii* ACICU. However, there were some instances of phylogenetic intermingling between integrases associated with *dusA* and non-*dusA* targets, indicating that phylogenetic analysis of DAIs is not alone sufficient to predict GEI specificity. Nonetheless, these non-cognate integrases may have diverged due to mutation of key amino acid residues that affect integrase site specificity (59).

### Detection of *dusA*-associated GEI excision

**Figure 4.**
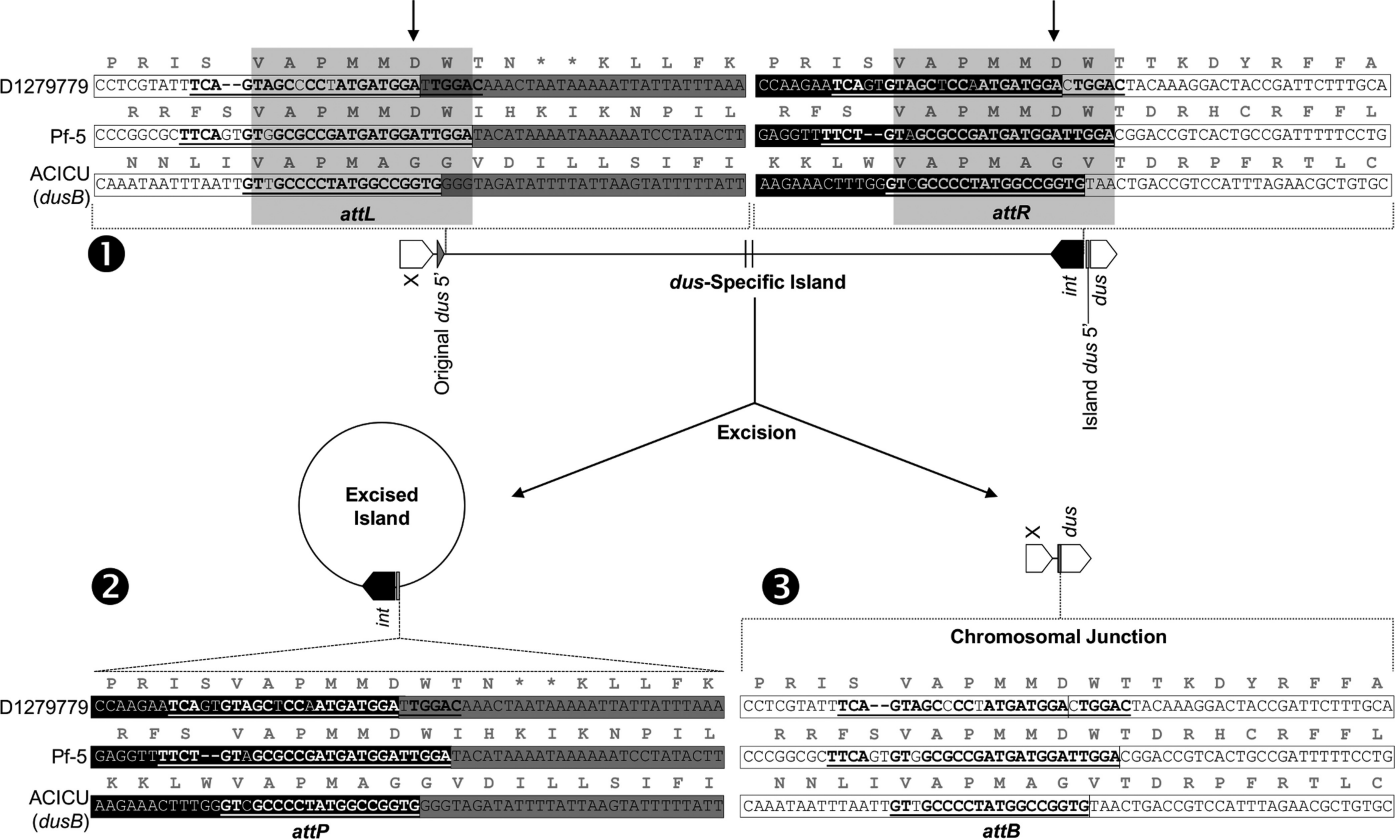
Predicted mechanism of GEI excision in *Acinetobacter baumannii* D1279779, *A. baumannii* ACICU and *Pseudomonas protegens* Pf-5, as a closed circular molecule (2) from the chromosome (1), resulting in the reformation of the native *dusA/B* sequence (3). The chromosomal gene flanking the island is designated as X (white). The shading of the nucleotide sequences represent their origin as either from the circularized GEI (black and dark grey) or chromosome (white). The underlined sequences represent the putative attachment sites involved in GEI excision and integration (*attP* and *attB*), which upon the latter occurring, a pair of imperfect direct repeats (*attL* and *attR*) are generated. Nucleotides in bold are identical in the *attP*/*attB* and *attL*/*attR* pairs of their respective organisms. The shaded (light grey) boxes represent the 21 bp consensus *attL*/*attR* sequences present in *dusA*-specific GEIs, whilst the indicated adenines (arrows) represent the experimentally determined island termini in the sequence of *Acinetobacter baumannii* D1279779.

Various types of GEIs have been previously demonstrated capable of excision from the chromosomes of bacteria (60,61), hence we investigated the possibility that the *dusA*-associated GEIs could also excise from the chromosomes of *A. baumannii* ACICU (*dusB*), *A. baumannii* D1279779 and *P. protegens* Pf-5. Oligonucleotides were designed to generate PCR amplicons only when the GEIs have excised as a covalently closed circular molecule, and the *dusA*/*dusB* chromosomal junction had reformed without a GEI inserted (Figures 2–4). When genomic DNA was isolated from cultures grown in MH media, neither of these amplicons were detectable unless ‘unconventional’ PCR protocols involving either a two-step amplification process of 35 cycles each or a single-step amplification with 60 cycles (62,63) were employed (data not shown). Similar PCR protocols were required for detection of other circularized GEIs (60,64).

MMC is a potent DNA-damaging agent which has been previously shown to induce the excision of various mobile elements, including integron cassettes (65), prophages and prophage remnants (66), and other GEIs (37). We hypothesized that treatment with MMC might increase the rate of excision of the *dus*-associated GEIs and facilitate their detection by PCR. The three strains were cultured with subinhibitory concentrations of MMC and total genomic DNA was isolated. Both the excised GEI and remnant *dusA/dusB* junction were detectable using a ‘standard’ 35 cycle PCR amplification in all three strains (Figure 2). In the case of *A. baumannii* D1279779, however, isolation of DNA using a kit-based derivative of the alkaline lysis method, conventionally used to isolate plasmid DNA, was required to enable detection of the excised GEI through PCR.

The amplicons obtained were consistent with the occurrence of GEI excision and the reformation of the wild-type *dusA* sequence. However, the amplicons generated were quite faint, despite MMC induction, suggesting an excision frequency of <10^−7^ (Figure 2). DNA sequencing had confirmed the occurrence of GEI excision, and the resultant sequence data was used to map both the GEI coordinates and putative attachment sites.

### The *dusA*-associated GEIs target a conserved region of *dusA*

Sequence data obtained from the amplicons of excised GEIs and reformed *dusA*/*dusB* genes from *A. baumannii* ACICU, *A. baumannii* D1279779 and *P. protegens* Pf-5 were examined to determine the precise island termini and the putative attachment sites involved in the integration and excision of the *dusA*-associated GEIs. This analysis revealed a semiconserved pair of direct repeats that formed the basis of the chromosomal and island attachment sites (Figure 4). The 5′ sequence of the *dusA* genes in these organisms were found to be derived from the *dusA*-associated GEIs inserted into this gene, whilst the original *dusA* 5′ end had unified into the wild-type *dusA* sequence upon GEI excision (Figures 3 and 4). On the basis of this sequence data, the *dusA*-associated GEIs will henceforth be referred to as *dusA*-specific GEIs.

The size of the putative island attachment sites in *A. baumannii* D1279779 and *P. protegens* Pf-5 varied from 26 to 28 bp, due to the presence of an additional GT dinucleotide in the island attachment site of D1279779 and the chromosome of Pf-5 (Figure 4). The 19 bp attachment sites of the *A. baumannii* ACICU *dusB*-specific GEI did share some commonality with attachment sites of the *dusA*-specific islands (Figure 4), despite its integrase targeting an otherwise non-cognate site. Like the *dusA*-specific GEIs, the *dusB*-specific GEI does provide a potential alternative 5′ end for the *dusB* gene (Supplementary Figure S4C). Examination of predicted attachment sites in other non-cognate sites in the pseudouridylate synthase (*rluA*) and aminobenzoyl-glutamate transporter (*abgT*) genes revealed only minor commonality in target sequence (Supplementary Figure S4A).

**Figure 5.**
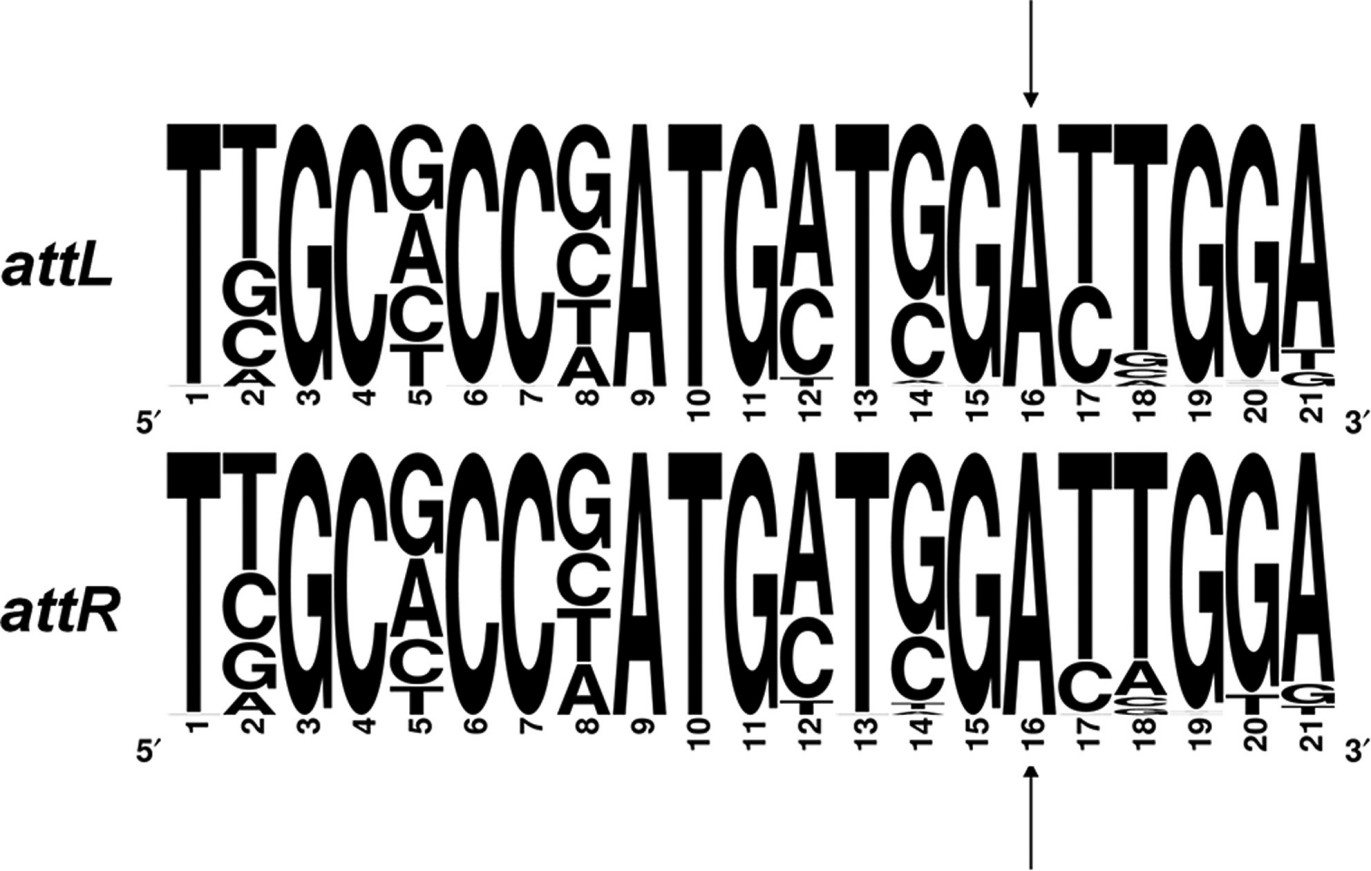
Logo consensus sequence of *attL* and *attR* direct repeats (as defined in Figure 4) generated with WebLogo (33), using alignments of flanking sequences from the 94 *dusA*-specific genome island sequences outlined in Supplementary Table S1. The indicated adenines (arrows) in both sequences represent the experimentally determined island termini in the sequence of *Acinetobacter baumannii* D1279779.

Prediction of the D1279779 and Pf-5 attachment sites facilitated the identification of direct repeats in the sequences of 92 other *dusA*-specific GEIs (Supplementary Table S1), originating from genome sequences with a sufficient level of completeness. It was found that all the examined GEIs were flanked by two 21 bp semiconserved attachment sequences (Figure 5), suggesting that despite the variability of the integrase protein sequence, these *dusA*-specific integrases target a conserved region within the *dusA* gene. The conserved sequence of the attachment sites allowed the genomic coordinates of GEIs in numerous organisms to be approximated.

### The *dusA*-specific islands are genetically and dimensionally variable

Determination of the aforementioned attachment sites facilitated revision of the size and genomic coordinates of *dus*-specific GEIs in *A. baumannii* and *P. protegens* Pf-5 (Table 2), as well as the discovery of 92 other *dusA*-specific GEIs (Supplementary Table S1).

**Table 2.** The size, genomic coordinates and putative phenotypes of *dus*-associated GEIs in *Acinetobacter* and *Pseudomonas* strains used in this study

Organism	Island name	Accession version	Genomic coordinates	Size (bp)	GC%	Predicted phenotype
*A. baumannii* D1279779	D1279779_RGP05	CP003967.2	595655..642997	47343	37.3	Type I restriction-modification, AST pathway
*A. baumannii* ACICU	pA_ICU_30 (Phage 04)	CP000863.1	2878594..2932506	53913	39.7	Phage lysogen
*P. protegens* Pf-5	Prophage 03	CP000076.1	2206875..2248336	41462	58.9	P2-like phage lysogen

Island name refers to that used previously in the literature. AST: arginine succinyltransferase.

Other than the *A. baumannii* D1279779 *dusA*-specific GEI (Figure 3), the size and integration sites of the other two GEIs in this study were not accurately predicted beforehand. The prophage pA_ICU_30 (43) was discovered to be 293 bp larger than previously predicted, consistent with the *dusB* target site being unidentified. However, Prophage 03 was discovered to be 7903 bp larger than previously predicted (45), which was consistent with both the *dusA* target site and several cargo genes being overlooked. The *hsdS* gene in D1279779_RGP05 (Figure 3), encoding the specificity subunit of a type I restriction-modification system, was on the basis of the published sequence thought to contain a frameshift whilst retaining some functionality (23). However, we confirmed through PCR and sequencing that this frameshift was the result of a sequencing error, and the genome sequence has been updated accordingly (GenBank Accession: CP003967.2).

Investigation of the 94 *dusA*-specific GEI sequences revealed extensive diversity in both the size and cargo gene content of these islands. The sizes of these GEIs ranged from 4758 bp in *Sinorhizobium fredii* USDA 257 to 184 808 bp in *Caulobacter* sp. K31, and averaged 42.6 kb in size (Supplementary Table S1). A 94-way reciprocal BLASTP search, conducted on the predicted proteome of all 94 *dusA*-specific islands, revealed that of the combined 4464 protein coding genes (excluding *dusA*), 2300 genes (51.5%) were unique to any one GEI. Only the integrase gene was common to all *dusA*-specific GEIs, though a gene encoding a *prtN* homologue was found in 60 of these islands (63.8%). PrtN regulates the production of pyocin in *Pseudomonas aeruginosa* (67), with the latter thought to be ancestrally derived from bacteriophages (68), though the role this particular gene might play in the regulation of these GEIs is not known.

Further examination of cargo gene function in *dusA*-specific GEIs revealed that about half of these islands were lysogenic prophages (48/94 islands), whilst the remainder consisted of GEIs and phage remnants (46/94 islands) (Supplementary Table S1). These GEIs encoded an assortment of putative functions including resistance to heavy metals, DNA restriction-modification, DNA replication, conjugative transfer and various metabolic enzymes (Supplementary Table S1). Several unique and noteworthy gene clusters were observed in some *dusA*-specific GEIs, including those encoding a Type IV secretion system in *Phenylobacterium zucineum* HLK1 (69), cytochrome biogenesis in *Mesorhizobium opportunistum* WSM2075 and the pyrimidine utilization (*rut*) pathway in *Acinetobacter baumannii* Naval-57. The variability of cargo gene content in the *dusA*-specific islands may be facilitated by the accretion and amelioration of additional MGEs, as suggested by the presence of other integrases and insertion sequences in some islands.

### The D1279779_RGP05 island does not bestow any detectable metabolic functions

We utilized a recently published protocol, hypothetically capable of deleting very large genomic regions in Gram-negative bacteria (38), to delete a 48.3 kb region in *A. baumannii* D1279779, encompassing the entire *dusA*-specific GEI, the *dusA* gene and the remnant *dusA* 5′ end, to generate *A. baumannii* D1279779Δ*dusA*. D1279779_RGP05 could not be selectively deleted to restore the original *dusA* sequence, as the remnant FRT site would otherwise disrupt the *dusA* gene. This effort represents the first successful generation of an unmarked, large chromosomal deletion in *A. baumannii*.

The isogenic variants of D1279779Δ*dusA* were complemented with *dusA* from D1279779 or *A. baumannii* ATCC 17978, which does not encode a *dusA*-specific island. We sought to determine the contribution of D1279779_RGP05 and *dusA* to the metabolic capabilities of *A. baumannii* D1279779 using PMs. Loss of *dusA*, and unexpectedly D1279779_RGP05, were not associated with any changes in carbon utilization (PM1-2) or osmotic, ionic and pH sensitivity (PM9-10). It is possible that the metabolic genes encoded in this island are functionally redundant, with equivalent functions encoded within the core genome of D1279779, or they may serve niche-specific functions that are not identifiable under the conditions of the PM assays.

### Integration of the *dusA*-specific islands may restore the functioning of DusA

Most *dusA*-associated GEIs and prophages were found to be specific for the 5′ end of *dusA*, characteristically replacing the 5′ end of *dusA* with new sequence provided by the GEI itself. Thus insertion of the GEI could affect either transcription of the *dusA* gene or the functioning of the DusA enzyme.

Analysis of the sequence upstream of *dusA* and *dusB*, prior to and after island integration, revealed the presence of putative −10 and −35 promoter regions in both instances (Supplementary Figure S4C). This suggested that both genes were still able to be transcribed, despite disruption by GEI and prophage integration. Examination of the protein coding sequence of DusA (and DusB) indicated that none of the amino acid residues in the affected region were known to be involved in either the binding of the flavin mononucleotide cofactor or tRNA in crystallized DUSs of *Thermotoga maritima* (70), *Thermus thermophilus* (71) and *E. coli* (72). However, the affected sequences do contain a structural motif potentially important for the overall functioning of the DusA enzyme.

DUSs contain a triosephosphate isomerase (TIM) barrel (70), one of the commonest protein folds, consisting of the canonical eightfold repeated beta–alpha unit (73). The wild-type N-terminal amino acid sequence of *dusA*, prior to the insertion of the *dusA*-associated GEI, encodes a beta strand (β-1) that forms part of the TIM barrel; the loss of this beta strand would presumably abolish DusA function. However, sequence-structure comparison revealed the new 5′ *dusA* sequence resulting from the GEI insertion provided a suitable replacement beta strand (Supplementary Figure S4B).

## CONCLUDING REMARKS

GEIs are classically associated with particular tRNA and tmRNA genes (9,10) but *dusA* presents an intriguing target of MGEs, as DusA is coincidentally involved in the post-transcriptional modification of tRNAs. However, of the more than 80 characterized tRNA-modifying enzymes in Proteobacteria (74), only a few have been reported as being targeted by mobile elements, including the Enterobacterial tRNA modification GTPase (*thdF*/*mnmE*) by *Salmonella* genomic island 1 (SGI1) (60), the *Polaromonas naphthalenivorans* CJ2 MiaB-like tRNA modifying enzyme (*rimO*) (75) and tRNA-pseudouridylate synthase (*rluA*) of *Acinetobacter* sp. ANC 3789 identified in this study.

The previously demonstrated dispensability of *dusA* in three independent deletion libraries (76–78), and the simultaneous dispensability of the *dusA*, *dusB* and *dusC* genes in *E. coli*, resulting in abolition of all dihydrouridine in this organism (79), suggested this gene would be a suitable target for MGE insertions. However, our bioinformatic analysis proposed that the functional and sequence integrity of *dusA*/DusA is maintained, due to replacement sequence provided by integration of these islands. The simulated laboratory conditions that these deletion libraries were constructed in may conceal the fitness cost that the loss of *dusA* would have on these (and other organisms) in their respective niches.

This supposition is reinforced by the discovery of DUS family enzymes involved in roles other than post-transcriptional reduction of uridine. The *dusA* gene of *Neisseria meningitidis* was upregulated during prolonged interaction with human epithelial cells, and when inactivated resulted in significant reduction to epithelial cell adherence (80). A gene encoding a DUS in *Clostridium botulinum* was downregulated during heat shock stress (81). Inactivation of *dusB* has been implicated in decreased biofilm formation in *Pseudomonas aeruginosa* (82) and *Actinobacillus pleuropneumoniae* (83). Downregulation of *dusC* has been observed during *E. coli* cell death mediated by both bactericidal antibiotics (84) and *Bdellovibrio bacteriovorus* predation (85).

The presence of dihydrouridine itself in RNA, one of the most abundant modified nucleobases in prokaryote and eukaryote tRNAs (86), has been demonstrated to alter its thermodynamic properties, resulting in greater conformational flexibility and dynamic motion (87). Adjustments in these thermodynamic properties are thought to be required for the maintenance of tRNA conformational flexibility in cryogenic conditions, as supported by the overabundance of this modified nucleobase in the tRNAs of psychrophilic microorganisms, compared to those present in psychrotrophic, mesophilic and thermophilic environments (88,89).

The *dusA*-specific integrases are phylogenetically distinct from previously characterized tyrosine recombinases, particularly those present in other laterally transferred regions, with the Rci protein family being the closest characterized relative. On the basis of tyrosine recombinase phylogeny, the *dusA*-specific GEIs can be considered distinct integrative elements (11). The *dusA*-specific GEIs are capable of excision as a circularized intermediate and consist of a highly mosaic cargo gene pool, encoding genes devoted to varying functions, such as GEI maintenance, propagation and replication, heavy metal resistance and augmentation of metabolic pathways. However, the only element unifying these otherwise disparate GEIs is the gene encoding the DAI.

## SUPPLEMENTARY DATA

Supplementary Data are available at NAR Online.

SUPPLEMENTARY DATA
