# Supplementary material for: A novel family of integrases associated with prophages and genomic islands integrated within the tRNA-dihydrouridine synthase A (dusA) gene
Source: Nucleic Acids Res. 2015 Apr 16;43(9):4547–57. doi: 10.1093/nar/gkv337 (PMC4482086; doi:10.1093/nar/gkv337)
Supplement: SUPPLEMENTARY DATA [file supp_43_9_4547__index.html]

A novel family of integrases associated with prophages and genomic islands integrated within the tRNA-dihydrouridine synthase A (dusA) gene — SUPPLEMENTARY DATA 

# A novel family of integrases associated with prophages and genomic islands integrated within the tRNA-dihydrouridine synthase A (*dusA*) gene

## SUPPLEMENTARY DATA

**Files in this Data Supplement:**

- SUPPLEMENTARY DATA
- SUPPLEMENTARY DATA
- SUPPLEMENTARY DATA
- SUPPLEMENTARY DATA
- SUPPLEMENTARY DATA
- SUPPLEMENTARY DATA
- SUPPLEMENTARY DATA
