# Supplementary material for: A novel family of integrases associated with prophages and genomic islands integrated within the tRNA-dihydrouridine synthase A (dusA) gene
Source: Nucleic Acids Res. 2015 Apr 16;43(9):4547–57. doi: 10.1093/nar/gkv337 (PMC4482086; doi:10.1093/nar/gkv337)
Supplement: SUPPLEMENTARY DATA [file supp_gkv337_nar-02196-h-2014-File008.pdf]

TABLE S1

Organism	Accession Version	Island Name	Coordinates	Size (bp)	G+C (%)	Locus Tag	CDS	*dusA*	*int*	*prtN*	Putative genomic island function(s)
Acidovorax sp. KKS102	NC_018708.1		2635879..2647205	11327	61.6	C380	12130-12220	12220	12195		Phage lysogen
Acinetobacter baumannii AB0057	NC_011586.1	$G08_{abn}$	686242..709787	23546	37.7	AB57	0641-0666	0641	0644	0643	Copper resistance
Acinetobacter baumannii AYE	NC_010410.1	$G08_{aye}$	3240071..3263741	23671	37.7	ABAYE	3198-3233	3223	3221	3220	Copper resistance
Acinetobacter baumannii D1279779	NC_020547.2	D1279779_RGP05	595655..642997	47343	37.3	ABD1	05270-05690	05270	05280	05290	Type I restriction-modification, arginine succinyltransferase pathway
Acinetobacter baumannii Naval-57	AMFP01000033.1		158876..205485	46610	39.2	ACINNAV57	0588-0630	0588	0590	0589	Type I restriction-modification, pyrimidine utilisation pathway
Acinetobacter baumannii OIFC137	AFDK01000002.1		3406689..3448088	41400	37.8	ACIN3137_A	3137-3319	3319	3320	3321	Copper and arsenic resistance
Acinetobacter guillouiae MSP4-18	ASQG01000039.1		23019..61244	38226	39.1	L291	2552-2589	2552	2553	2554	Arsenic, chromium and heavy metal resistance
Acinetobacter gyllenbergii MTCC 11365	ASQH01000014.1		31583..54268	22686	39.2	L293	1364-1388	1364	1365		Arsenic and heavy metal resistance, ferrous iron transport
Acinetobacter lwoffii SH145	NZ_GG705066.1		22884..55777	32894	40.0	HMPREF0017	02541-02567	02541	02542	02543	Type I restriction-modification, potassium transport
Acinetobacter radioresistens SK82	NZ_ACVR01000072.1		64274..94100	29827	40.4	ACIRA0001	0075-0114	0114	0113	0109	Phage lysogen
Acinetobacter sp. NBRC100985	NZ_BAEB01000006.1		68257..94432	26172	36.2	ACT4_006	00690-00890	00690	00700	00710	Type II restriction-modification, DNA helicase
Acinetobacter sp. WC-743	AMFQ01000090.1		137442..162473	25032	38.3	ACINWC743	3553-3576	3553	3554		Arsenic resistance, ferric iron uptake, metabolism
Agrobacterium sp. H13-3	NC_015183.1		1437021..1478152	41132	56.6	AGROH133	06152-06219	06152	06153		Phage lysogen
Aliivibrio salmonicida LFI1238	NC_011312.1	ϕ VS1	428180..437090	8911	33.9	VSALI	0388-0399	0399	0398		Phage remnant
Alteromonas macleodii Balearic Sea AD45	NC_018679.1		3848848..3868705	19858	38.6	AMBAS45	16335-16445	16335	16340		Phage remnant
Alteromonas macleodii str. 'Deep ecotype' (Island 1)	NC_011138.2		3651333..3677013	25681	41.0	MADE	1016775-1016910	1016775	1016780		Type I restriction-modification, IS*Ama4* transposase
Alteromonas macleodii str. 'Deep ecotype' (Island 2)	NC_011138.2		3721852..3747532	25681	41.0	MADE	1017135-1017275	1017275	1017270		Type I restriction-modification, IS*Ama4* transposase
Bradyrhizobium sp. BTAi1	NC_009485.1	Island 10	3464601..3604950	140350	62.7	BBta	3310-3456	3456	3455	3310	Conjugal transfer, cationic metal resistance, DNA replication and maintenance
Burkholderia cenocepacia H111	CAFQ01000015.1		36888..83895	47008	61.3	I35	1589-1653	1589	1590		Phage lysogen
Burkholderia gladioli BSR3	NC_015381.1		2106016..2128524	22509	57.0	bgla_1g	18670-18900	18670	18690	18680	Phage lysogen
Caulobacter sp. K31	NC_010338.1		1999331..2184138	184808	66.5	Caul	2043-1864	2043	2042		Conjugal transfer, carbonaceous compound degradation, multiple integrases
Colwellia psychrerythraea 34H	NC_003910.7		364208..384624	20417	36.1	CPS	0353-0383	0353	0354	0369	Phage remnant, IS*Cps1* transposase
Escherichia coli 101-1	NZ_AAMK02000006.1		51324..79125	27802	50.8	EC1011	3356-3393	3393	3392	3358	Phage lysogen
Escherichia coli 3003	AFAF02000002.1		362630..409709	47080	50.9	EC3003	4528-4592	4592	4591	4530	Phage lysogen
Escherichia coli B171	NZ_AAJX02000004.1		51511..95437	43927	50.5	EcB171	0688-0730	0730	0729	0670	Phage lysogen
Escherichia coli ETEC H10407	NC_017633.1		4684277..4723914	39638	50.0	ETEC	4359-4304	4359	4358	4308	Phage lysogen
Escherichia coli KTE41	ASUF01000002.1		42719..83413	40695	51.0	WGC	00045-00108	00108	00107	00048	Phage lysogen
Escherichia coli O157H7 str. EC4115	NC_011353.1		5179273..5190127	10855	46.1	ECH74115	5530-5551	5551	5550	5532	Phage remnant
Escherichia coli PA40	AKLQ01000063.1		54348..65206	10859	46.1	ECPA40	5484-5503	5503	5502	5485	Phage remnant
Escherichia coli STEC H.1.8	AFDY01000049.1		31089..83492	52404	52.8	ECSTECH18	5069-5153	5153	5152	5071	Phage lysogen, IS3 transposase, tRNAs genes
Glaciecola mesophila KMM 241	BAEP01000023.1		158489..187213	28725	40.2	GMES	1194-1222	1222	1221		Type I restriction-modification
Herbaspirillium sp. CF444	NZ_AKJW01000015.1		60763..69441	8679	50.7	PMI16	00553-00564	00564	00563		Phage remnant
Hoeflea phototrophica DFL-43	NZ_ABIA02000005.1		330659..363334	32676	57.4	HPDFL43	10257-10387	10257	10262		Metabolism
Hyphomonas neptunium ATCC 15444	NC_008358.1		269653..283801	14149	55.4	HNE	0283-0293	0293	0292		Unknown
Kingella oralis ATCC 51147	ACJW02000002.1		776377..811527	35151	53.7	GCWU000324	01205-01259	01259	01258	01207	Phage lysogen
Labrenzia alexandrii DFL-11	NZ_EQ973121.1		523596..529163	5568	47.6	SADFL11	1138-2029	1138	2278		Degraded genomic island
Mesorhizobium opportunistum WSM2075	NC_015675.1		4428933..4582578	153646	59.9	Mesop	4302-4443	4443	4442		Conjugal transfer, cytochrome biogenesis
Neisseria meningitidis 73696	APTR01000030.1		40652..84499	43848	51.9	NM73696	0415-0353	0415	0414		Phage lysogen
Neisseria meningitidis ATCC 13091	NZ_AEEF01000001.1		40927..82652	41726	51.1	HMPREF0602	0052-0118	0118	0117		Phage lysogen
Pandoraea sp. SD6-2	AQOU01000038.1		40667..49678	9012	56.5	C266	17541-17581	17541	17546	17581	Unknown
Paracoccus denitrificans PD1222	NC_008686.1		1388784..1523382	134599	61.8	Pden	1420-1548	1420	1421		Conjugal transfer
Parvibaculum lavamentivorans DS-1	NC_009719.1		1096727..1154718	57992	58.9	Plav	1004-1050	1050	1049		Pseudoazurin production, nitrogen oxide metabolism
Phaeobacter gallaeciensis DSM 17395	NZ_ABIF01000015.1		17762..54815	37054	54.4	RGBS107	16878-16743	16743	16748		Metabolism
Phenylobacterium zucineum HLK1	NC_011144.1		2656855..2791880	135026	67.7	PHZ_c	2343-2442	2343	2344		Type IV secretion system, various metabolic enzymes
Pseudoalteromonas tunicata D2	NZ_AAOH01000003.1		381923..420459	38537	43.9	PTD2	08004-08264	08264	08259	08009	Phage lysogen
Pseudogulbenkiania sp. NH8B	NC_016002.1		558446..601013	42568	62.8	NH8B	0534-0598	0598	0597	0589	Phage lysogen
Pseudomonas aeruginosa MSH-10	ASWW01000003.1		839103..894227	55125	62.1	LE46	1516-1588	1516	1517	01518	Phage lysogen
Pseudomonas aeruginosa PA7	NC_009656.1	RGP56	2411812..2468822	57011	62.8	PSPA7	2362-2435	2362	2363	2364	Phage lysogen
Pseudomonas chlororaphis O6	NZ_AHOT01000027.1	Prophage 3	770994..829360	58367	59.0	PchlO6	2091-2179	2091	2093	2092	Phage lysogen
Pseudomonas fluorescens BRIP34879	AMZW01000089.1		14881..50242	35362	56.3	A986	15166-15361	15166	15171	15176	Phage lysogen
Pseudomonas fluorescens Q2-87	AGBM01000001.1	Prophage 2	2130087..2168671	38585	58.7	PflQ2	3552-3598	3598	3596	3597	Phage lysogen
Pseudomonas fulva 12-X	NC_015556.1		2257605..2363077	105473	58.8	Psefu	2114-2205	2114	2115	2116	Conjugal transfer, DNA replication and maintenance
Pseudomonas protegens CHA0	NC_021237.1		2253742..2290680	36939	58.4	PFLCHA0_c	20300-20720	20300	20310	20320	Phage lysogen
Pseudomonas protegens Pf-5	CP000076.1	Prophage 03	2206875..2248336	41462	58.9	PFL	1975-2023	1975	1976		Phage lysogen
Pseudomonas pseudoalcaligenes KF707	AJMR01000228.1		412242..525826	113585	61.8	ppKF707	4047-4141	4047	4048		Conjugal transfer, amino acid metabolism, DNA replication and partitioning
Pseudomonas putida GB-1	NC_010322.1		1936916..1981735	44820	60.9	PputGB1	1710-1763	1710	1711	1712	Phage lysogen
Pseudomonas putida H8234	CP005976.1		1761316..1814709	53394	60.2	L483	08090-08470	08090	08095	08100	Phage lysogen
Pseudomonas putida S16	NC_015733.1		1947645..1989806	42162	59.2	PPS	1736-1780	1736	1737	1739	Phage lysogen
Pseudomonas resinovorans NBRC 106553	NC_021499		3872818..3943813	70966	62.1	PCA10	35880-35170	35880	35870	35850	Arsenic resistance, metabolism
Pseudomonas sp. GM60	NZ_AKJI01000047.1		90198..107498	17301	52.8	PM132	02372-02396	02396	02395	02394	Phage remnant
Pseudomonas sp. GM74	AKJG01000046.1		100576..146826	46251	57.0	PMI34	01891-01945	01891	01892	01894	Phage lysogen
Pseudomonas sp. GM80	NZ_AKJD01000246.1		17402..64255	46854	58.1	PMI37	05674-05731	05731	05730	05729	Phage lysogen
Pseudomonas sp. P179	AQFO01000014.1		388699..441269	52571	61.5	HMPREF1224	06117-06055	06117	06116	06115	Phage lysogen
Pseudomonas synxantha BG33R	NZ_CM001514.1		1852442..1863492	11051	52.2	PseBG33	1689-1699	1689	1691	1690	Unknown
Pseudomonas syringae pv. avellanae str. ISPaVe037	AKCK01000055.1		118856..135069	16214	50.7	Pav037	1760-1772	1772	1771		Phage remnant
Rhizobium lupini HPC(L)	AMQQ01000004.1		165839..208803	42965	56.3	C241	02694-02994	02694	02699		Phage lysogen, tRNA-Met
Rhodobacteraceae bacterium KLH11	NZ_DS999531.1		2330944..2379161	48218	55.4	RKLH11	310-1236	310	1807	3025	Phage lysogen
Rhodomicrobium vannielii ATCC 17100†	NC_014664.1		1918261..1967577	49317	60.3	Rvan	1748-1799	1748	1749	1797	Conjugal transfer, various transposases
Ruegeria sp. R11	NZ_DS999054.1		541627..590903	49277	56.7	RR11	404-1812	1812	3187		Phage lysogen
Shewanella baltica OS155	NC_009052.1		4210997..4253441	42445	46.0	Sbal	3585-3633	3585	3586	3620	Phage lysogen
Shewanella baltica OS185	NC_009665.1		866082..908384	42303	46.2	Shew185	0722-0774	0774	0773	0734	Phage lysogen

Organism	Accession Version	Island Name	Coordinates	Size (bp)	G+C (%)	Locus Tag	CDS	*dusA*	*int*	*prtN*	Putative genomic island function(s)
Shewanella baltica OS195	NC_009997.1		903068..946900	43833	45.3	Sbal195	0751-0806	0806	0805	0763	Phage lysogen
Shewanella baltica OS223	NC_011663.1		883717..926065	42349	46.1	Sba1223	0742-0799	0799	0798	0759	Phage lysogen, IS*Sba2* tranposase
Shewanella denitrificans OS217	NC_007954.1		3867007..3909462	42456	45.3	Sden	3232-3281	3232	3233	3273	Phage lysogen
Shewanella sp. MR-7	NC_008322.1		820506..853673	33168	48.6	Shewmr7	0699-0746	0746	0745	0703	Phage lysogen
Sinorhizobium fredii HH103	HE616890.1		1772042..1814196	42155	62.0	SFHH103	01636-01689	01636	01637	01689	Phage lysogen
Sinorhizobium fredii USDA 257	NC_018000.1		4359830..4364587	4758	57.5	USDA257_c	41740-41790	41740	41750		Degraded genomic island, DNA ligase
Sinorhizobium medicae WSM419	NC_009636.1		1717430..1768028	50599	61.2	Smed	1621-1683	1621	1622	1680	Phage lysogen
Sinorhizobium meliloti AK83	NC_015590.1		1828582..1877761	49180	60.8	Sinme	1811-1876	1811	1812	1873	Phage lysogen
Sinorhizobium meliloti BL225C	CP002740.1		1728034..1776517	48484	61.2	SinmeB	1656-1716	1656	1657	1714	Phage lysogen
Sinorhizobium meliloti SM11	CP001830.1		2406519..2454942	48424	60.9	SM11_chr	1426-1491	1491	1490	1426	Phage lysogen
Starkeya novella DSM 506	NC_014217.1		882664..907567	24904	66.5	Snov	0848-0872	0872	0871		Alkanesulphonate metabolism
Variovorax paradoxus S110	NC_012791.1		2202985..2265954	62970	64.1	Vapar	2059-2122	2122	2121		DNA replication
Vibrio cholerae O1 str. EM-1676A	APFY01000090.1		11544..48041	36498	42.7	VCEM1676A	000425-000456	000456	000455		Fimbrial biogenesis
Vibrio cholerae TMA 21	NZ_ACHY01000008.1		9867..38075	28209	43.9	VCB	000225-000242	000225	000226	000241	Conjugal transfer
Vibrio mimicus VM223	NZ_ADAJ01000005.1		215734..227834	12101	46.6	VMA	000316-000303	000316	000315		Phage remnant, DNA replication
Vibrio parahaemolyticus BB22OP	NC_019955.1		2860513..2893508	32996	49.8	VPBB	2545-2586	2545	2546	2550	Phage lysogen
Vibrio shilonii AK1	NZ_ABCH01000029.1		6437..42147	35711	43.5	VSAK1	13606-13831	13606	13611	13636	Phage lysogen
Vibrio sinaloensis DSM 21326	NZ_AEVT01000002.1		63274..77654	14381	45.9	VISI1226	20525-20665	20665	20660	20540	Phage lysogen
Vibrio sp. 16	DS999366.1		11141..26033	14893	47.0	VPMS16	1054-1081	1081	1080	1074	Unknown
Vibrio sp. RC341	NZ_ACZT01000016.1		6993..19073	12081	46.3	VCJ	000612-000632	000612	000613		Phage remnant, DNA replication
Vibrio splendidus 12B01	NZ_AAMR01000034.1		7414..21480	14067	44.0	V12B01	10660-10775	10660	10665	10760	Phage remnant, DNA replication
Xanthomonas albilineans GPE PC73	NC_013722.1		219708..271808	52101	59.9	XALc	0171-0242	0242	0241		Phage lysogen
Xenorhabdus bovienii SS-2004	NC_013892.1		3797963..3840279	42317	48.3	XBJ1	3925-3991	3925	3926	3991	Phage lysogen, tRNA-Phe

†: Secondary *attR* site at 1987629..1987644
