# Supplementary material for: A novel family of integrases associated with prophages and genomic islands integrated within the tRNA-dihydrouridine synthase A (dusA) gene
Source: Nucleic Acids Res. 2015 Apr 16;43(9):4547–57. doi: 10.1093/nar/gkv337 (PMC4482086; doi:10.1093/nar/gkv337)
Supplement: SUPPLEMENTARY DATA [file supp_gkv337_nar-02196-h-2014-File009.pdf]

Oligonucleotide	Sequence (5'→3')	Size (bp)
D1279779_Excison_FOR	GAGAAGCTTAGGAAATCAACGACAG	740
D1279779_Excison_REV	TCTATGGCATCTGACAAAGTTAGGTC	
D1279779_Junction_FOR	ACTTCGTCATAACCCCAGTCTT	494
D1279779_Junction_REV	GTGGGACTCGTTGTATTTTGTA	
ACICU_Excison_FOR	ATGTAGCATTATGGCTGAAGAG	534
ACICU_Excison_REV	GCTAGTGATTTCTTACTGAATGTCT	
ACICU_Junction_FOR	TAACTCTCCATCAAAATTAGCAC	534
ACICU_Junction_REV	TGTCTAAGTCTTTCATAATGTCATC	
PF5_Excison_FOR	TATCTGAAGAGAGGGCAGAATA	472
PF5_Excison_REV	CTTTCCTGATAAACTGTGACG	
PF5_Junction_FOR	CACCGATCATGTTGTTCTGCAC	485
PF5_Junction_REV	ATACTCGGACAACCTCGCTAGAC	
RGP05_UP_FOR[†]	AGATTATT**GGATCC**TTAATGAGTGGC	947
RGP05_UP_REV[†]	CGATTGAAGTGA**GAGCTC**AACCGATA	
RGP05_DWN_FOR[†]	GAACACCAA**GGATCC**GCCAGCCCATG	965
RGP05_DWN_REV[†]	CTCTATCTTT**TCCGGA**AGTTTTACTGG	
P1_DAMGE_FOR	AGCTCAAGTGCTTATTCCTCT	50324
P4_DAMGE_REV	ATCTTACCTGCCAATGTAAAAC	
FRT-T7F	AAATTAATACGACTCACTATAGG	229
FRT-SP6R	TACGATTTAGGTGACACTATAG	
FRT-leftF	AATCCATCTTGTTCAATCATGC	N/A
FRT-rightR	AATTCGAGCTCGGGAAGATC	
Abaumannii_dusA_FOR	ATC**GGATCC**TAATTTTGAATATTTCGAGCC	1253
D1279779_dusA_REV	CCATT**GGATCC**ATTATGGTGTAAATTTTGG	
ATCC17978_dusA_REV	TCA**GGATCC**TATATTTAACATAAGGGC	1242
pWH1266_ColonyScreen_FOR	CTTCGCTACTTGGAGCCACTAT	152
pWH1266_ColonyScreen_REV	ATCTTCCCCATCGGTGATGT	

†: Creation of *Acinetobacter baumannii* D1279779 Δ*dusA* was conducted using oligonucleotides with two differing restriction sites, rather than one, to ensure correct directionality of FRT plasmid integration. While complete deletion of the *dusA*-specific genomic island in this organism was successful, the remnant 'scar' that had formed consisted of the multiple cloning site of pKFRT/FLP, rather than the expected FRT sequence.
