# Supplementary material for: A novel family of integrases associated with prophages and genomic islands integrated within the tRNA-dihydrouridine synthase A (dusA) gene
Source: Nucleic Acids Res. 2015 Apr 16;43(9):4547–57. doi: 10.1093/nar/gkv337 (PMC4482086; doi:10.1093/nar/gkv337)
Supplement: SUPPLEMENTARY DATA [file supp_gkv337_nar-02196-h-2014-File010.pdf]

FIGURE S1

```
                                                  10        20        30        40        50        60
                                          ....|....|....|....|....|....|....|....|....|....|....|....|
Agrobacterium sp. H13-3                   ------------------------------------------------------------ 1
Bradyrhizobium sp. BTAi1                  ------------------------------------------------------------ 1
N. gonorrhoeae FA 1090                    ------------------------------------------------------------ 1
B. gladioli BSR3                          -------------------------------------MGSITV-------RKRKDGSAAY 16
A. baumannii D1279779                     -------------------------------------MGTIVP-------RKRKDGSIGY 16
P. protegens Pf-5                         -------------------------------------MGTITA-------RKRKDGSVGY 16
S. enterica Typhimurium R64               -------------------------------------MCAQTARLSDRQLKAVKPKDKDY 23
S. sonnei P9 plasmid ColIb-P9             MAVRGFLLQTSTSDHQWKQPPIWGSFGGFAKHPLQTPPRHQHMALTDLKVRTAKPAEKQQ 60
E. coli ED1a                              ------------------------------------------MPLSDIQVRNLKPREKAY 18
Salmonella genomic island 1               ------------------------------------------------------------ 1
Haemophilus phage HP1                     ------------------------------------------------------------ 1
Enterobacteria phage λ                    ------------------------------------------------------------ 1
S. bongori CIEM46082                      ------------------------------------------------------------ 1
H. influenzae 1056                        ------------------------------------------------------------ 1
P. syringae pv. phaseolicola              ---MKVSVNKRNPNSKGLQQL---------------------RLVYYYGVVEGEDGKKRA 36
Y. pseudotuberculosis 32777               -----MAVRKDTKNGKW---L---------------------AEVYVN----GNASRKWF 27
A. baumannii AB0057                       ------------------------------------------------------------ 1
S. sonnei                                 ------------------------------------------------------MGRRRS 6
K. pneumoniae                             ------------------------------------------------------------ 1
H. influenzae Rd KW20                     ------------------------------------------------------------ 1
E. coli K12                               ------------------------------------------------------------ 1
P. naphthalenivorans CJ2                  ------------------------------------------------------------ 1
B. cenocepacia PC184                      -------------------------------------MGTITA-------RKRKDGTTAH 16
Enterobacter sp. 638                      -------------------------------------MATITK-------RRNPSGETVY 16
P. putida F1                              -------------------------------------MGSITA-------RKGADGNVSY 16
Shewanella sp. MR-7                       -------------------------------------MGRSTNKLTAKAVLNAKAEAKPY 23
L. vestfoldensis SKA53                    -------------------------------------MARITRPLTNNEILKAKPREKDF 23

                                                  70        80        90       100       110       120
                                          ....|....|....|....|....|....|....|....|....|....|....|....|
Agrobacterium sp. H13-3                   ------------------------------------------------------------ 1
Bradyrhizobium sp. BTAi1                  ------------------------------------------------------------ 1
N. gonorrhoeae FA 1090                    ------------------------------------------------------------ 1
B. gladioli BSR3                          ------TAQIRIMQKG----VTVYQESQTF----------DRKTTAQAWIRKREAE-LHE 55
A. baumannii D1279779                     ------TAQIRLKVKG----KVVHTEAKTF----------DREPAASAWIKKRERE-LSQ 55
P. protegens Pf-5                         ------TAQILRKKGG----RIVFREAKTF----------DRKREAEAWVRFRETE-IDK 55
S. enterica Typhimurium R64               VLTDGDGLQLRVRVNRSMQWNFNYRHPVTKNRINMALGSYPEVSLAQARRKAVEAREVLA 83
S. sonnei P9 plasmid ColIb-P9             KLYDGSGLLLLITPAGGKRWIFKYRIDG--KEKSLALGTYPDISLAEARSRRDSAREKLA 118
E. coli ED1a                              KVSDFEGLFVLVKPNGSKLWQFKYRMDG--KERLLSIGVYPNISLAQARKTKDGARANVA 76
Salmonella genomic island 1               ------------------------------------------------------------ 1
Haemophilus phage HP1                     ------------------------------------------------------------ 1
Enterobacteria phage λ                    ------------------------------------------------------------ 1
S. bongori CIEM46082                      ------------------------------------------MKQ--------------- 3
H. influenzae 1056                        ----------------------------------------M-HPQ--------------- 4
P. syringae pv. phaseolicola              -KRDYEPLE-----------LYLYENPKTQAERQHNKE--M-LRQAEA---------ARS 72
Y. pseudotuberculosis 32777               -LTKGDALR-------------FYNQAKEQTT--------------SA---------VDS 50
A. baumannii AB0057                       ------------------------------------------------------------ 1
S. sonnei                                 HERRDLPPNLYIRNNG----YYCYRDPRTGKEFGLGRD--RRIAITEA---------IQ- 50
K. pneumoniae                             ------------------------------------------------------------ 1
H. influenzae Rd KW20                     ------------------------------------------MKTAT------------- 5
E. coli K12                               -----------------------------------MNR--Y-NRNDK------------- 9
P. naphthalenivorans CJ2                  ------------------------------------------------------------ 1
B. cenocepacia PC184                      ------LAQVLIKRGG----AILHREARTF----------DRKQAAAAWLERREKE-LAA 55
Enterobacter sp. 638                      ------RVQVRVGKKG----YPAFNESRTF----------SKKALAVEWGKKREAE-IEA 55
P. putida F1                              ------RAAIRINKKG----YPAYSESKTF----------YSKKVAENWLKKREVE-IQE 55
Shewanella sp. MR-7                       RLSDGGNLYLYVRTAG-KTWEFRYIRPSTSKPTYFGLGSYPDVSLAEARDKALEARKNLA 82
L. vestfoldensis SKA53                    TLHDGDGLFLLVKTSGKKLWRFRYQRPNSTSRTNLSLGAYPALTLAAARLIRDQHLSLLA 83
```

```
                                       130       140       150       160       170       180
                                ....|....|....|....|....|....|....|....|....|....|....|....|
Agrobacterium sp. H13-3         ----MFR-------------KIKIRKMTLNRALDKYLKTVSIHKKGHLQEFYRVNVIKRH 43
Bradyrhizobium sp. BTAi1        -----MP-------------SPRIRKMSLSRALDKYLKTVSVHKKGHQQEFYRSNVIKRY 42
N. gonorrhoeae FA 1090          -----MP-------------SPRIRKMSLSRALDKYLKTVSVHKKGHQQEFYRSNVIKRY 42
B. gladioli BSR3                PGAI--E-------------RANRSGVSVKEMIDQYLKQYEKLRPLGKTKRATLNAIKES 100
A. baumannii D1279779           PGAI--E-------------GAKREDPTLGEVIARYIRED--KRGIGRTKKQVLETIRGK 98
P. protegens Pf-5               PGAL--E-------------RLNANRFTLADAIDRYVKE---KGTMGATKDQVLRTIKTF 97
S. enterica Typhimurium R64     QGIDP-KAQRND----LAQAKLAETEHTFEKVASAWFELKKDSVTPAYAEDIWRSLTLHV 138
S. sonnei P9 plasmid ColIb-P9   AGLDP-SEAKKA----DKRAAQLAAASSFEIVAREWFETQRGGWSEVYAGKVINCLEVDV 173
E. coli ED1a                    AGIDP-SEAKQQ----EKRQRREVNDQTFEKLGAEFFAKQRKEGKSAATLSKTEYHLQLA 131
Salmonella genomic island 1     -----------------------MKPMTLPELTQEYILTHDLRPDTVKIYKAATKAYVNF 37
Haemophilus phage HP1           ---------------------------------------------------------MQH 3
Enterobacteria phage λ          ---------------------METNITWQQLIDEYFFAKPLRSASEWSYTKVFKSFVHY 38
S. bongori CIEM46082            ---DL-ARI------------EQFLDALWLE--------KNLAENTLNAYRRDLSMMVEW 39
H. influenzae 1056              ---SQ-SSI------------DAFIDALWLE--------DGLSRNTLAAYRRDLSLYAGW 40
P. syringae pv. phaseolicola    ARLVE-SHSNKFQLEDRVKLASSFY--DYYDK-----LTASKESGSSSNYSIWISAGKHL 124
Y. pseudotuberculosis 32777     VQVLE-SS-------DL--PALSFYVQEWFDL-----HGKTLSDGKAR-----LAKLKNL 90
A. baumannii AB0057             ------------------MEIEMNKANYDEILQDYFFSKSLRPATEWSYRKVINSFRRY 41
S. sonnei                       ANIELFSGHKHKPLTARINSDNSVTLHSWLDRYEKILASRGIKQKTLINYMSKIKAIR-- 108
K. pneumoniae                   ------MSN------------SPFLNSIRTDM-----RQKGYALKTEKTYLHWIKRFILF 37
H. influenzae Rd KW20           APLPP-LRS------------VKVLDQLRERI-----RYLHYSLRTEQAYVNWVRAFIRF 47
E. coli K12                     PDWVP-PRS------------IKLLDQVRERV-----RYLHYILQTEKAYVYWAKAFVLW 51
P. naphthalenivorans CJ2        ---ML-TAL------------NRYWDYLRIE--------RQMSPHTITNYQHQLDATIKI 36
B. cenocepacia PC184            PGGL--E-------------QEKGLDPVLSDVIERYVSES--KKEIGRTKAQVLNAIKGY 98
Enterobacter sp. 638            GPELLFK-------------RGKVKMMTLSEAMRKYLNET--L-GAGRSKKMGLRFLMEF 99
P. putida F1                    NPDILFG-------------KEQLIDLTLSDAIDKYLDEV--GSEYGRTKRYALLLIKKL 100
Shewanella sp. MR-7             EGIDP-QLLKVE----NKLKAATENAQTLKHIAQMWMDSKQGSIKPKTIEGNWRKLELYA 137
L. vestfoldensis SKA53          QDIDP-QQQQEI----VSEQRQIKLDSVFSTVAANWFQLKSKSVTPDYAKDIWRSLDKDV 138

                                       190       200       210       220       230       240
                                ....|....|....|....|....|....|....|....|....|....|....|....|
Agrobacterium sp. H13-3         ------PMAERYMDEITTVDIATYRDQRLAQINP----RTGRQITGNTVRLELALLSSLF 93
Bradyrhizobium sp. BTAi1        ------PIALRNMDEITTVDIATYRDVRLAEINP----RTGKPITGNTVRLELALLSSLF 92
N. gonorrhoeae FA 1090          ------PIALRNMDEITTVDIATYRDVRLAEINP----RTGKPITGNTVRLELALLSSLF 92
B. gladioli BSR3                ------WLGDVTDAELTSQKLVEYAVWR----------METFGIQAQTVGNDLAHLGAVL 144
A. baumannii D1279779           ------DIAERPCSELRSADYIQFARSL--------------DVQPQTVGNYMSHLGAIV 138
P. protegens Pf-5               ------NLATMDCSDIRSDDIVTFANEL------------AEGRKPQTVGSYISHLSSIF 139
S. enterica Typhimurium R64     F--P--SMKSTPISEVSAPMVIKILRPIES------------KGSLETVKRLSQRLNEIM 182
S. sonnei P9 plasmid ColIb-P9   F--P--RLGARPIASIDAPELLAIIRTVES------------RGVRETAKRVLQRSRAVF 217
E. coli ED1a                    S--R--DFGRKPIIEITAPMILKTLRKVEA------------KGHYETAHRLRSRIGSIF 175
Salmonella genomic island 1     F-----GECLA--CETTHRDMLEWRRSELA------------RISKRSWNTYSSHLRTVY 78
Haemophilus phage HP1           N-----CNLKYP-DEVSKLLILQWRKAVVG----------K-SIIEVTWNSYVRQLKTIF 46
Enterobacteria phage λ          M-----GPLSCP-NDVTYHKVLAWRRFLLK----------EKKLSGRTWNNKVAHMRAIF 82
S. bongori CIEM46082            LHHRGLTLA-----TAQSDDLQALLAERLE----------GGYKATS-SARLLSAVRRLF 83
H. influenzae 1056              LHAKEQQHRKL--DDTDEDDLKAYFSFR-H----------AATKATS-ANRCLTVFKRYF 86
P. syringae pv. phaseolicola    R--SYHGRAELTFEEIDKKFLEGFRKYLLEEPLTKSQSKLA----KNTASSYFNKVRAAL 178
Y. pseudotuberculosis 32777     C--SNLG--DPPANEFNAKIFADYRKRRLDGEFSVNKNNPPKEATVNREHAYLRAVFNEL 146
A. baumannii AB0057             I-----GDNLLP-GEVDRLTVLNWRRHVLN----------KQGLSSITWNNKVAHMRAIF 85
S. sonnei                       -----RGLPDAPLEDITTKEIAAMLNGYID----------EGKAAS--AKLIRSTLSDAF 151
K. pneumoniae                   -----HKKRHP--QTMGSEEVRLFLSSLAN----------SRHVAINTQKIALNA----L 76
H. influenzae Rd KW20           -----HGVRHP--ATLGSSEVEAFLSWLAN----------ERKVSVSTHRQALAA----L 86
E. coli K12                     TARSHGGFRHP--REMGQAEVEGFLTMLAT----------EKQVAPATHRQALNA----L 95
P. naphthalenivorans CJ2        LAQQDIHS--W--TQVTPSVVRFILAESKK----------QGLKEKS-LALRLSALRRFL 81
B. cenocepacia PC184            ------EIAQRRCSTIGSTDLVEFANQL------------VSKVQPQTVSNYLSHLAAIF 140
Enterobacter sp. 638            ------PIGGIGIDKLKRSDFAEHVMQRRRGIPE----LDIAPIAASTALQELQYIRSVL 149
P. putida F1                    ------PIARNIITKIHSTHLAEHVALRRRGVPN----LGLEPIATSTQQHELLHIRGVL 150
Shewanella sp. MR-7             F--P--KLGSIAISRLTAPIAIAALKPLEQ------------KGHLETVKRTAQLLNEIM 181
L. vestfoldensis SKA53          F--P--AIGEIPVQEIKARTIIEALEPIKA------------RGALETVRRLVQRINEIM 182
```

```
                                          250       260       270       280       290       300
                                 ....|....|....|....|....|....|....|....|....|....|....|....|
Agrobacterium sp. H13-3          NIASVEWGTCRM--------NP-VELVRKPK-----ISSGRDRRLTSGEERRLSRYFRDK 139
Bradyrhizobium sp. BTAi1         NIARVEWGTCRT--------NP-VELVRKPK-----VSSGRDRRLTSSEERRLSRYFREK 138
N. gonorrhoeae FA 1090           NIARVEWGTCRT--------NP-VELVRKPK-----VSSGRDRRLTSSEERRLSRYFREK 138
B. gladioli BSR3                 SVARPAWGYDVDPHAMSDARSV-LRKMGAVS-----RSRERNRRPTLDELDRILTYFEQM 198
A. baumannii D1279779            RIARPAWGYPLAESEFDDAMVV-GKRLGLTG-----KSVARDRRPTPDELNRILEYYTEM 192
P. protegens Pf-5                SIARPAWGMPLDPVAIRDAQTV-LRKLNTIA-----NSESRTRRPTLAELDKIMEYFTKR 193
S. enterica Typhimurium R64      TYGVNS-----GM--I--FANP-LSGIRAVF---KKPKKENMAALPPEELPELMLEIA-- 227
S. sonnei P9 plasmid ColIb-P9    QYGIMT-----GR--C--ARNP-AADIDAETVLKKSTGVQHMARVKVTEIPQLMRDID-- 265
E. coli ED1a                     RYAVAS-----GI--A--ETDP-TYALRDAL--IRPTRKHRAAIIDPQALGRLMNEID-- 221
Salmonella genomic island 1      RYAMEH-----GL--VELKVNP-LKDT-RVM-----PTKRPKKTIGNDVIVRARNWLRFL 124
Haemophilus phage HP1            KFGIEN-----QF--LPFTKNP-FDGL-FIR-----EGKRKRKVYSPSDLDRLSFGIK-- 90
Enterobacteria phage λ           NYGIQR-----GL--LQYDENP-FNNS-VVK-----PDKKRKKTLTQAQIEYAYQIMEQY 128
S. bongori CIEM46082             QYLYRE-----KF--R--EDDP-SAHLASPK-----LPQRLPKDLSEAQVERLLQAPL-- 126
H. influenzae 1056               RWALRE-----NL--I--AADP-TLKLQSAK-----QALRVPKVMSEAQVDALLAAPD-- 129
P. syringae pv. phaseolicola     NEAFRE-----GI--I--RDNP-VQRVKSVK-----AENTQRTYLTLDEVRAMTKAEC-- 221
Y. pseudotuberculosis 32777      KS--LR-----KW--T--TENP-LDGVRLFK-----ERETELAFLYERDIYR-LLAEC-- 186
A. baumannii AB0057              NHALLH-----DL--VSFKNNP-FNGV-IVR-----PDVKRKKTLTQSEIKKIYLIMEAR 131
S. sonnei                        REAIAE-----GH--I--TTNH-VAATRAAK------SEVRRSRLTADEYLKIYQAAE-- 193
K. pneumoniae                    AFLYNR-----FL--Q--QPLGDIDYIPAS------KPRRLPSVISANEVQRILQV---- 117
H. influenzae Rd KW20            LFFYGK-----VL--C--TDLPWLQEIGRPR-----PSRRLPVVLTPDEVVRILGF---- 128
E. coli K12                      LFLYRQ-----VL--G--MELPWMQQIGRPP-----ERKRIPVVLTVQEVQTLLSH---- 137
P. naphthalenivorans CJ2         SFLVQQ-----GE--L--KVNP-ATGISAPK-----QGRHLPKNMDGEQVQQLLA-ND-- 123
B. cenocepacia PC184             AIARPAWGYPLDQAAMKDAFVV-AKRLGITS-----KSRTRERRPSLAELDLLMQHFGER 194
Enterobacter sp. 638             KHAFYVWGLEIGWQELDFAANG-LKRSNMVA-----KSAIRDRLPTTEELQTLTTYFLRQ 203
P. putida F1                     SHASVMWGMDIDLSSFDKATAQ-LRKTRQIS-----SSKVRDRLPTNEELVTLTKFFAER 204
Shewanella sp. MR-7              NYSVNS-----GY--I--HANP-LSGIREVF---RKSKVEHLKALKPAELAELLQTVA-- 226
L. vestfoldensis SKA53           IYAVNT-----GL--L--DANP-ASGVGMAF---ERPKKQNMLTLRPEELPKLMRSIG-- 227

                                          310       320       330       340       350       360
                                 ....|....|....|....|....|....|....|....|....|....|....|....|
Agrobacterium sp. H13-3          NQQL--------------------YVIFHLALETAMRQGEILTLRWEHLDL-QH--GVAH 176
Bradyrhizobium sp. BTAi1         NLML--------------------YVIFHLALETAMRQGEILALRWEHIDL-RH--GVAH 175
N. gonorrhoeae FA 1090           NLML--------------------YVIFHLALETAMRQGEILALRWEHIDL-RH--GVAH 175
B. gladioli BSR3                 RDRR------------R--QEIDMLRVIVFALFSTRRQEEITRIRWDLLNE-SE--QSAL 241
A. baumannii D1279779            AKRE------------R--AELPMRELIVFALFSTRRQEEITTIRVEDFE---G--DRVL 233
P. protegens Pf-5                NQAT------------P--HVSRMDRVVAFAIYSTRRQEEIVRIEWEDLDE-VH--SRIL 236
S. enterica Typhimurium R64      ----------------NASIKRTTRCLIEWQLHTMTRPAEAATTRWVDIDF-ERRVWTIP 270
S. sonnei P9 plasmid ColIb-P9    ----------------EYSGDLVTRLALRFMALTFVRTKEMIQAEWPEIDV-GAAEWRVP 308
E. coli ED1a                     ----------------VFEGQATTRIALKLLAMVAQRPGEIRHAKWSEIDF-VKKVWSIP 264
Salmonella genomic island 1      VQEE----LSTGKRSEITPAWFWLA-VFETFYYTGIRLNALLCLRYENVDL-GQRLIRVR 178
Haemophilus phage HP1            --------ESKYLPAILRPLWFTRA-LIMTFRYTAIRRSQLNKLRIRDIDL-LNQVIHIS 140
Enterobacteria phage λ           ENQENTGLGLKYSRCALFPAWFWLT-VLDTLYYTGIRQNQLLHIRLNDVDL-REGQIRLI 186
S. bongori CIEM46082             ---------------IDQPLELRDKAMLEVLYATGLRVSELVGLTMSDISL-RQ--GVVR 168
H. influenzae 1056               ---------------DDTPLGLRDRAMLELMYASGLRVSELVGLKTFHVGL-NE--GALR 171
P. syringae pv. phaseolicola     ---------------RY----DVLKRAFLFSCTTGLRWSDIQKLTWKEIEEFQDGHYRII 262
Y. pseudotuberculosis 32777      ---------------DNS-RNPDLGLIVRICLATGARWSEAETLTQSQVMP-----YKIT 225
A. baumannii AB0057              EREEHVGIM-GKSRSALRPAWFWLT-VVDTLRYTGMRQNQLLHIRLGDVNL-NDGWINLR 188
S. sonnei                        ---------------S---SPCWLRLAMELAVVTGQRVGDLCEMKWSDIVD-----GYLY 230
K. pneumoniae                    -------------------MDTRNQVIFTLLYGAGLRINECLRLRVKDFDF-DN--GCIT 155
H. influenzae Rd KW20            -------------------LEGEHRLFAQLLYGTGMRISEGLQLRVKDLDF-DH--GTII 166
E. coli K12                      -------------------MAGTEALLAALLYGSGLRLREALGLRVKDVDF-DR--HAII 175
P. naphthalenivorans CJ2         ---------------SKEPIDIRDRAILELMYSSGLRLSELQGLDLNSINT-RV--REVR 165
B. cenocepacia PC184             QQRR------------P--SSVPMQKVIAFAIFSTRRLEEITRLRRADLDE-VG--SKIL 237
Enterobacter sp. 638             WQSR------------K--SSIPMHLIMWLAIYTSRRQDEICRLLFDDWHK-ND--CTRP 246
P. putida F1                     WKLN------------KYGTKYPMHLVIWFAIFSCRREAELTRLWLQDYDS-YH--SSWK 249
Shewanella sp. MR-7              ----------------NANLLLTTRCLIEWQLHTMTRPNEAAGARWEEIDF-DAKLWTIP 269
L. vestfoldensis SKA53           ----------------MSNLSVPTRCLIELQLLTLVRPSEASGARWAEIDI-DAKLWKIP 270
```

```
                                                370       380       390       400       410       420
                                       ....|....|....|....|....|....|....|....|....|....|....|....|
Agrobacterium sp. H13-3                LP-----ETKNGLPRDVPLSRKA---RNYLQILPQQ-----------ING---------- 207
Bradyrhizobium sp. BTAi1               LP-----ETKNGHSRDVPLSRRA---RNFLQMMPVN-----------LHG---------- 206
N. gonorrhoeae FA 1090                 LP-----ETKNGHSRDVPLSRRA---RNFLQMMPVN-----------LHG---------- 206
B. gladioli BSR3                       VTDMKNPGQKYGNDVWCHMPDEA---WRVLQSMPKV------------AD---------- 276
A. baumannii D1279779                  VRDMKHPGQKKGNDTWCDVPPEA---ARVIEAVRPK------------SG---------- 268
P. protegens Pf-5                      VRDLKHPGQKKGNDVWCEIPPEA---MQIIKAMPKN------------GP---------- 271
S. enterica Typhimurium R64            PE-----RMKKSRPHSIPLSDQA-------MSLLEIL--KSHSGHR-E------------ 303
S. sonnei P9 plasmid ColIb-P9          AE-----RMKMRDPHIVPLSRQA-------LDVLAQL--REINGQQ-R------------ 341
E. coli ED1a                           AD-----RMKMRRDHIVPLPDQA-------IALLDQL--RRMNGNG-E------------ 297
Salmonella genomic island 1            GE-----TEKTHREFMIPIPDGL---MPHLVLVMDTA--KKVGFSG-TDQ---------- 217
Haemophilus phage HP1                  PE-----INKNHEYHILPISHTL---YPYLDNLLNEL--KKMKQSA-DAQ---------- 179
Enterobacteria phage λ                 TE-----GCKNHKEHYVPVISFL---RPRLTCLVEKA--QSEGLKG-NDR---------- 225
S. bongori CIEM46082                   V------IGKGNKERLVPLGEEA---VYWLETYLEHG--RPWLLNG-VSI---------- 206
H. influenzae 1056                     V------MGKGSAERLVPFGQVA---REWIVRYIAES--RPAILGG-QQT---------- 209
P. syringae pv. phaseolicola           FK-----QAKLLNAGNSLVYLDL---PDSAVKLMG------------ERQ---------- 292
Y. pseudotuberculosis 32777            FT-----NTKSKKNRTVPISKEL----------FD------------MLP---------- 248
A. baumannii AB0057                    PE-----ASKNHKEHRIPIARVL---RPRLERLVATA--IEKGANQ-VDQ---------- 227
S. sonnei                              VE-----QSKTGVKIAIPTALHIDALGISMKETLDKC--KEILGGE-TII---------A 273
K. pneumoniae                          VH-----DGKGGKSRNSLLPTRL---IPAIKXLIEQA--RLIQQDD-NLQ-GVGPSLPFA 203
H. influenzae Rd KW20                  VR-----EGKGSKDRALMLPESL---APSLREQLSRA--RAWWLKD-QAEGRSGVALPDA 215
E. coli K12                            VR-----SGKGDKDRVVMLPRAL---VPRLRAQLIQV--RAVWGQD-RATGRGGVYLPHA 224
P. naphthalenivorans CJ2               V------IGKGNKERVVPFGRYA---SHAIQEWLKVR-----ALFN-PKD---------- 200
B. cenocepacia PC184                   VRDMKNPGEKLGNDVWCDLPAEA---LHVAQSMPSD------------TD---------- 272
Enterobacter sp. 638                   VRDLKNPNGSTGNNKEFDILPMA---LPVIDELPEESVRKRMLANKGIAD---------- 293
P. putida F1                           VHDLKNPNGSKGNHKSFEVLEPC---KTIVELLLDNEVRSRMLQLGYDER---------- 296
Shewanella sp. MR-7                    ES-----RMKMNREHIIPLTEHT-------LAILETV--KPIGEHS-Q------------ 302
L. vestfoldensis SKA53                 AE-----RMKAKREHIVPLSPQA-------LEILEIM--TPISAHR-E------------ 303

                                                430       440       450       460       470       480
                                       ....|....|....|....|....|....|....|....|....|....|....|....|
Agrobacterium sp. H13-3                ---------------NVFSYT--------------SSGFKSAWRTALLD----LKIE--N 232
Bradyrhizobium sp. BTAi1               ---------------NVFDYT--------------ASGFKNAWRIATQR----LRIE--D 231
N. gonorrhoeae FA 1090                 ---------------NVFDYT--------------ASGFKNAWRIATQR----LRIE--D 231
B. gladioli BSR3                       ---------------EVFPYN--------------SRSVSASFTRACNF----LEIE--D 301
A. baumannii D1279779                  ---------------PIFPYN--------------HRSISASFTKACAF----LSID--D 293
P. protegens Pf-5                      ---------------RIFPYG--------------TAGVGAAFTRACQF----LEIE--D 296
S. enterica Typhimurium R64            ---------------YVFPADR----------NPRTHANSQTANMALKR----MGFQD-R 333
S. sonnei P9 plasmid ColIb-P9          ---------------FVFYSVQ----------GR-SHISNNTMLYALYR----MGYKS-R 370
E. coli ED1a                           ---------------YLFPSLR----------TWKRPMSENTLNAALRR----MGYSGDE 328
Salmonella genomic island 1            ----------------VFNINRFSGHYKR--EYMNSDQVEAMYKKLTNM----TG---TR 252
Haemophilus phage HP1                  ----------------LFNINLFSKAVKKRRGKEMTADQISYLFKVISKH----TG---VN 216
Enterobacteria phage λ                 ----------------LFNIALFTGKDPAIGDDMDSPQVRAFFRRLSKE----CQ---FA 262
S. bongori CIEM46082                   -------------------------D-VLFPSQRAQQMTRQTFWHRIKHYAVLAGIDSEK 240
H. influenzae 1056                     -------------------------D-DLFVTGHGHGMSRVMFWMLVKKYALLAGIHS-P 242
P. syringae pv. phaseolicola           ---------------------------DKAERVFKGLKYSSYTNVALLHWAMLAGVQ-KH 324
Y. pseudotuberculosis 32777            ---------------------------KKRGRLFN-DAYESFENAVLRA---EIELP-KG 276
A. baumannii AB0057                    ----------------LFNISRIDGRKETVTENMDSPPLRSFFRRLSVE----CR---CT 264
S. sonnei                              STRREP--------------------------LSS-GTVSRYFMRARKASGL--SFEGDP 304
K. pneumoniae                          LDHKYPSAYRQAAWMFVFPSSTLCNH-PYNGKLCRHHLHDSVARKALKAAVQKAGIVSKR 262
H. influenzae Rd KW20                  LERKYPRAGHSWPWFWVFAQHTHSTD-PRSGVVRRHHMYDQTFQRAFKRAVEQAGIT-KP 273
E. coli K12                            LERKYPRAGESWAWFWVFPSAKLSVD-PQTGVERRHHLFEERLNRQLKKAVVQAGIA-KH 282
P. naphthalenivorans CJ2               -------------------------E-ALFVSQLGNRISHRAIQKRLETWGIRQGLNS-H 233
B. cenocepacia PC184                   ---------------EIFPYT--------------TDAIGMGFTRACQL----LGIV--D 297
Enterobacter sp. 638                   ---------------SLVPCN--------------GKSVSAAWTRACKV----LGIK--D 318
P. putida F1                           ---------------LLLPLN--------------PKSIGKEFRDACKM----LGIE--D 321
Shewanella sp. MR-7                    ---------------FVFPSSK----------DPKKHTDPETINKALGR----MGLKG-R 332
L. vestfoldensis SKA53                 ---------------YVFPSRN----------DPKQPMNSQTANAAIKR----IGYGG-R 333
```

```
                                                490       500       510       520       530       540
                                       ....|....|....|....|....|....|....|....|....|....|....|....|
Agrobacterium sp. H13-3                LHFHDLRHEAISRFFELGTLNVMEVAAISGHRSLN-MLKRYTHLRAYQL-------VSKL 284
Bradyrhizobium sp. BTAi1               LHFHDLRHEAISRFFELGSLNVMEIAAISGHRSMN-MLKRYTHLRAWQL-------VSKL 283
N. gonorrhoeae FA 1090                 LHFHDLRHEAISRFFELGSLNVMEIAAISGHRSMN-MLKRYTHLRAWQL-------VSKL 283
B. gladioli BSR3                       LHFHDLRHDGVSRLFEMG-WDIPKVASVSGHRDWN-SMRRYTHLRGNGDPYAGWQWIERV 359
A. baumannii D1279779                  LHFHDLRHEGASRLFEMG-LNIPHVAAVTGHRSWS-SLKRYTHLRHVGDRWARWAWLDRV 351
P. protegens Pf-5                      LHFHDLRHEGISRLFEMG-RTIPLAASVSGHRTWN-SLKRYTQIRERGDKFEGWKWLKTV 354
S. enterica Typhimurium R64            LVSHGMRSMASTILNEHG-WDPELIEVALAHVDKDEVRSAYNRADYIERRRPMMAW---- 388
S. sonnei P9 plasmid ColIb-P9          MTGHGFRGLAATTLRELG-YSRDVVERQMAHAERNQVTAAYVHAEYLPERRKMMQH---- 425
E. coli ED1a                           MTAHGFRASFSTLANESGLWNPDAIERALAHVEKNEVRRAYARGEHWEERVRLANW---- 384
Salmonella genomic island 1            MTPHRFRHTIASELMRQPERNIHITKNLLNHSNIA-TTMEYIEPDYDLMREVMNERGQQQ 311
Haemophilus phage HP1                  SSPHRFRHTAATNLMKNPE-NLYVVKQLLGHKDIK-VTLSYIESDISSLRKHIDCL---- 270
Enterobacteria phage λ                 ISPHRFRHTLATEMMKMPEQNLHMAQSVLGHSNMK-STLEYVENDIAVMGRALEAQFMQI 321
S. bongori CIEM46082                   LSPHVLRHAFATHLLNHGA-DLRVVQMLLGHSDLS-TTQIYTHVATERL----RQLHQQ- 293
H. influenzae 1056                     LSPHTLRHAFATHLLNHGA-DLRAVQMLLGHADIS-TTTIYTHVARERL----KSIHAE- 295
P. syringae pv. phaseolicola           VTFHVGRHTFAVAQLNRGV-DIYSLSRLLGHSELR-TTEIYADILESRR----VTAMRG- 377
Y. pseudotuberculosis 32777            QLTHVLRHTFASHFMMNGG-NILVLKEILGHSTIE-MTMRYAHFAPSHL----ESAVKF- 329
A. baumannii AB0057                    ISPHRFRHTIATEMMKSPDRNLKVVQTLLGHSSIA-VTLEYVEGDIDSLRLALEETFERK 323
S. sonnei                              PTFHELRSLS-ARLYEKQI-SDKFAQHLLGHKSDT-MASQYRDDRGREW----DKIEIK- 356
K. pneumoniae                          VTCHTFRHSFATHLLQAGR-DIRTVQELLGHNDVK-TTQIYTHVLGQHF----AGTTSP- 315
H. influenzae Rd KW20                  ATPHTLRHSFATALLRSGY-DIRTVQDLLGHSDVS-TTMIYTHVLKVGG----AASNGR- 326
E. coli K12                            VSVHTLRHSFATHLLQAGT-DIRTVQELLGHSDVS-TTMIYTHVLKVAA----GGTSSP- 335
P. naphthalenivorans CJ2               LNPHKLRHSFATHMLEASS-DLRAVQELLGHSNLS-TTQIYTHLNFQHL----AEVYDQ- 286
B. cenocepacia PC184                   LHFHDLRHDGVSRLFEMG-HNVPQVAAVSGHRSWS-SLKRYTHLRQTGNKYEGWKWLEVV 355
Enterobacter sp. 638                   LRFHDLRHEAATRMAEDG-FTIPQMQRVTLHDGWN-SLQRYVSVRKRSTRLDFKEAMMQA 376
P. putida F1                           LRFHDLRHEGCTRLAEQS-FTIPEIQKVSLHDSWS-SLQRYVSVKSRRNVIQLEEVLRLI 379
Shewanella sp. MR-7                    TTGHGLRSLASTTLNEQG-FDGDVIEAALAHVDKNQIRSAYDRTTYLERRTKLMEW---- 387
L. vestfoldensis SKA53                 LVAHGLRSIASTAMNEEG-FNPDVIEAALAHSDKNEVRRAYNRSTYLEARRELMDW---- 388

                                                550       560       570       580       590       600
                                       ....|....|....|....|....|....|....|....|....|....|....|....|
Agrobacterium sp. H13-3                DTKRKQTCKIAPYFVP-YPATVGNRNG----------------------L---------- 311
Bradyrhizobium sp. BTAi1               DARRRQTQKVAAWFVP-YPAHITTIDE----------------------ENGQ------- 313
N. gonorrhoeae FA 1090                 DARRRQTQKVAAWFVP-YPAHITTINE----------------------ENGQ------- 313
B. gladioli BSR3                       ISGPVIEAQVRV------------KRR----------------------AAGR------- 378
A. baumannii D1279779                  APLQEQS----------------------------------------------------- 358
P. protegens Pf-5                      TDE--------------------------------------------------------- 357
S. enterica Typhimurium R64            ------------WSEYILKASTGNLSASAMNV---ARDRNVVPIR--------------- 418
S. sonnei P9 plasmid ColIb-P9          ------------WADHLDELRAGAK---------------IIPITASTP----------- 447
E. coli ED1a                           ------------WAGYLENLQAM------------------------------------- 395
Salmonella genomic island 1            AK-------INYLVRPIIPKSSGPAAGSAVPRVSLVSGTELQPATTESSEAKKADDTASN 364
Haemophilus phage HP1                  ------------------------------------------------------------ 270
Enterobacteria phage λ                 KA-------AH--ARSIYS---GLTK----NR---------------------------- 337
S. bongori CIEM46082                   -------------HHPRA------------------------------------------ 298
H. influenzae 1056                     -------------HHPRG------------------------------------------ 300
P. syringae pv. phaseolicola           -----------------FPDIF---------------EDKVQESGTCCPHCGK---SVLN 402
Y. pseudotuberculosis 32777            -----------------NPLSN---------------P--AQ------------------ 337
A. baumannii AB0057                    EV-------F-------------------------------------------------- 326
S. sonnei                              ------------------------------------------------------------ 356
K. pneumoniae                          -------------ADGLMLLIN---------------Q---------------------- 325
H. influenzae Rd KW20                  -------------LRKVLPASA---------------DGRQQPVV------A-------- 344
E. coli K12                            -------------LDALALHLS---------------PG--------------------- 346
P. naphthalenivorans CJ2               -------------AHPRAK---------------------RKK----------------- 295
B. cenocepacia PC184                   TVKTAPG----------------------------------------------------- 362
Enterobacter sp. 638                   QS-DIKSGK--------------------------------------------------- 384
P. putida F1                           DE-T-------------------------------------------------------- 382
Shewanella sp. MR-7                    ------------WSNHIATAAVGSLSVTAGSR---KLGA--------------------- 411
L. vestfoldensis SKA53                 ------------WGSAIYK----------------------------------------- 395
```

```
                                          610       620       630       640       650       660
                                 ....|....|....|....|....|....|....|....|....|....|....|....|
Agrobacterium sp. H13-3          ----FIVTLHDFD-L-----------ETRAETRELAISHASVLLLRT------------- 342
Bradyrhizobium sp. BTAi1         KA--HRIEIGDFDNL-----------HVTATTKEEAVHRASEVLLRT------------- 347
N. gonorrhoeae FA 1090           KA--HRIEIGDFDNL-----------HVTATTKEEAVHRASEVLLRT------------- 347
B. gladioli BSR3                 AP---------------------------------------------------------- 380
A. baumannii D1279779            ------------------------------------------------------------ 358
P. protegens Pf-5                ------------------------------------------------------------ 357
S. enterica Typhimurium R64      ------------------------------------------------------------ 418
S. sonnei P9 plasmid ColIb-P9    ------------------------------------------------------------ 447
E. coli ED1a                     ------------------------------------------------------------ 395
Salmonella genomic island 1      PPIAERLELMSTAPLVNVLKSTPVSVHSEPATSNEAKAQALSLISDAPEMEYEFGELEDI 424
Haemophilus phage HP1            ------------------------------------------------------------ 270
Enterobacteria phage λ           ------------------------------------------------------------ 337
S. bongori CIEM46082             ------------------------------------------------------------ 298
H. influenzae 1056               ------------------------------------------------------------ 300
P. syringae pv. phaseolicola     KTL--------------------------------------------------------- 405
Y. pseudotuberculosis 32777      ------------------------------------------------------------ 337
A. baumannii AB0057              ------------------------------------------------------------ 326
S. sonnei                        ------------------------------------------------------------ 356
K. pneumoniae                    ------------------------------------------------------------ 325
H. influenzae Rd KW20            ------------------------------------------------------------ 344
E. coli K12                      ------------------------------------------------------------ 346
P. naphthalenivorans CJ2         ------------------------------------------------------------ 295
B. cenocepacia PC184             ------------------------------------------------------------ 362
Enterobacter sp. 638             ------------------------------------------------------------ 384
P. putida F1                     ------------------------------------------------------------ 382
Shewanella sp. MR-7              ------------------------------------------------------------ 411
L. vestfoldensis SKA53           ------------------------------------------------------------ 395

                                          670       680       690       700       710
                                 ....|....|....|....|....|....|....|....|....|....|
Agrobacterium sp. H13-3          -------------LAQAAQRGERVPTPGELPANIDARVMICPLTS----- 374
Bradyrhizobium sp. BTAi1         -------------LAIAAQKGERVPSPGALPVNDPDYIMICPLNPGSTPL 384
N. gonorrhoeae FA 1090           -------------LAIAAQKGERVPSPGALPVNDPDYIMICPLNPGSTPL 384
B. gladioli BSR3                 -------------------------------------------------- 380
A. baumannii D1279779            -------------------------------------------------- 358
P. protegens Pf-5                -------------------------------------------------- 357
S. enterica Typhimurium R64      -------------------------------------------------- 418
S. sonnei P9 plasmid ColIb-P9    -------------------------------------------------- 447
E. coli ED1a                     -------------------------------------------------- 395
Salmonella genomic island 1      ARWIRENAAGEMVVQIAAEEDIGTISEGKASIQSPDGYYRIPWSSGSVK- 473
Haemophilus phage HP1            -------------------------------------------------- 270
Enterobacteria phage λ           -------------------------------------------------- 337
S. bongori CIEM46082             -------------------------------------------------- 298
H. influenzae 1056               -------------------------------------------------- 300
P. syringae pv. phaseolicola     -------------------------------------------------- 405
Y. pseudotuberculosis 32777      -------------------------------------------------- 337
A. baumannii AB0057              -------------------------------------------------- 326
S. sonnei                        -------------------------------------------------- 356
K. pneumoniae                    -------------------------------------------------- 325
H. influenzae Rd KW20            -------------------------------------------------- 344
E. coli K12                      -------------------------------------------------- 346
P. naphthalenivorans CJ2         -------------------------------------------------- 295
B. cenocepacia PC184             -------------------------------------------------- 362
Enterobacter sp. 638             -------------------------------------------------- 384
P. putida F1                     -------------------------------------------------- 382
Shewanella sp. MR-7              -------------------------------------------------- 411
L. vestfoldensis SKA53           -------------------------------------------------- 395
```
