# Supplementary material for: A novel family of integrases associated with prophages and genomic islands integrated within the tRNA-dihydrouridine synthase A (dusA) gene
Source: Nucleic Acids Res. 2015 Apr 16;43(9):4547–57. doi: 10.1093/nar/gkv337 (PMC4482086; doi:10.1093/nar/gkv337)
Supplement: SUPPLEMENTARY DATA [file supp_gkv337_nar-02196-h-2014-File011.pdf]

FIGURE S2

```
                                    10        20        30        40        50        60
                            ....|....|....|....|....|....|....|....|....|....|....|....|
Agrobacterium sp. H13-3     MGTIT--ARKRKDGSVGYTAQILRKKGGRIVFREAKTFDRKREAEAWVRFRETEIDKPGA 58
Bradyrhizobium sp. BTAi1    MGTIT--ARKRKDGTTAHLAQVLIKRGGAILHREARTFDRKQAAAAWLERREKELAAPGG 58
P. denitrificans PD1222     MATISKLP----SGS----WRVQVRRKGH--Y-ISESFLRRGDAELWARTVEGKIDRGEP 49
B. gladioli BSR3            MGTIV--PRKRKDGSIGYTAQIRLKVKGKVVHTEAKTFDREPAASAWIKKRERELSQPGA 58
N. gonorrhoeae FA 1090      MATIT--KRRNPSGETVYRVQVRVGKKGYPAFNESRTFSKKALAVEWGKKREAEIEAGPE 58
V. paradoxus S110           MPTFKQLP----SGN----WRAQVRRKGV--Y-ASETFRRHKDAQEWALATERRIDLGEP 49
A. baumannii D1279779       MGSIT--ARKGADGNVSYRAAIRINKKGYPAYSESKTFYSKKVAENWLKKREVEIQENPD 58
P. protegens Pf-5           MGSIT--VRKRKDGSAAYTAQIRIMQKGVTVYQESQTFDRKTTAQAWIRKREAELHEPGA 58
S. baltica OS155            MASYSIQKREKADGTVRHRCLVRVKKNGKILYTEQRTFTKYAAAEAWGKDR--VIDIESN 58
S. enterica plasmid R64     ------------------------------------------------------------ 1
S. sonnei plasmid ColIb-P9  ------------------------------------------------------------ 1
E. coli ED1a                ------------------------------------------------------------ 1
Clustal Consensus                                                                        1

                                    70        80        90       100       110       120
                            ....|....|....|....|....|....|....|....|....|....|....|....|
Agrobacterium sp. H13-3     L--ERLNANRFTLADAIDRYVKE----KGTMGATKDQVLRTIKTFNLATMDCSDIRSDDI 112
Bradyrhizobium sp. BTAi1    L--EQEKGLDPVLSDVIERYVSE-S--KKEIGRTKAQVLNAIKGYEIAQRRCSTIGSTDL 113
P. denitrificans PD1222     I-H-TRVAAAKTFGDLIDLHRADLADVGRTLGRSKAASLDFLA-LRLGALKPAELGREKL 106
B. gladioli BSR3            I--EGAKREDPTLGEVIARYIRE-D--KRGIGRTKKQVLETIRGKDIAERPCSELRSADY 113
N. gonorrhoeae FA 1090      LLFKRGKVKMMTLSEAMRKYLNE-T--L-GAGRSKKMGLRFLMEFPIGGIGIDKLKRSDF 114
V. paradoxus S110           A-TRSKVKDPTTFGDLIDLHVTDMKEVLRAPRRSKAFTLDALN-TKLGKLKLKDLTRERL 107
A. baumannii D1279779       ILFGKEQLIDLTLSDAIDKYLDE-V--GSEYGRTKRYALLLIKKLPIARNIITKIHSTHL 115
P. protegens Pf-5           I--ERANRSGVSVKEMIDQYLKQ-YEKLRPLGKTKRATLNAIKESWLGDVTDAELTSQKL 115
S. baltica OS155            G-FATEDTAPITLGSIISKALTD-ENIDSSIGRSKRFCLRLLSDCDIAKLNLTDIKPHHI 116
S. enterica plasmid R64     --MPSPRIRKMSLSRALDKYLKT-VSVHKKGHQQEFYRSNVIKRYPIALRNMDEITTVDI 57
S. sonnei plasmid ColIb-P9  --MPSPRIRKMSLSRALDKYLKT-VSVHKKGHQQEFYRSNVIKRYPIALRNMDEITTVDI 57
E. coli ED1a                -MFRKIKIRKMTLNRALDKYLKT-VSIHKKGHLQEFYRVNVIKRHPMAERYMDEITTVDI 58
Clustal Consensus                       .   :                 :      :    :.      :      5

                                   130       140       150       160       170       180
                            ....|....|....|....|....|....|....|....|....|....|....|....|
Agrobacterium sp. H13-3     VTFANEL--------AEGRKPQTVGSYISHLSSIFSIARPAWGMPLDPVAIRDAQTVLRK 164
Bradyrhizobium sp. BTAi1    VEFANQL--------VSKVQPQTVSNYLSHLAAIFAIARPAWGYPLDQAAMKDAFVVAKR 165
P. denitrificans PD1222     VEFGRQRA-------KEGAGPVTVGIDLGYIKTILSHAAAVHGIIAPTEGVDLARIALTR 159
B. gladioli BSR3            IQFARSL----------DVQPQTVGNYMSHLGAIVRIARPAWGYPLAESEFDDAMVVGKR 163
N. gonorrhoeae FA 1090      AEHVMQRRRGIPELDIAPIAASTALQELQYIRSVLKHAFYVWGLEIGWQELDFAANGLKR 174
V. paradoxus S110           IQFGKDRA-------KEGAGPVTISMDIGYIKLVVSHAAAVHGVRVPVEPIDLARIALKR 160
A. baumannii D1279779       AEHVALRRRGVPNLGLEPIATSTQQHELLHIRGVLSHASVMWGMDIDLSSFDKATAQLRK 175
P. protegens Pf-5           VEYAVWR------METFGIQAQTVGNDLAHLGAVLSVARPAWGYDVDPHAMSDARSVLRK 169
S. baltica OS155            IDHCKLRR-------SAGTGPSTIAVDVSVIRWLLRIAKSNFGHEVSQISVIEAYDALYS 169
S. enterica plasmid R64     ATYRDVRLAEINPRTGKPITGNTVRLELALLSSLFNIARVEWGTCRTN------PVELVR 111
S. sonnei plasmid ColIb-P9  ATYRDVRLAEINPRTGKPITGNTVRLELALLSSLFNIARVEWGTCRTN------PVELVR 111
E. coli ED1a                ATYRDQRLAQINPRTGRQITGNTVRLELALLSSLFNIASVEWGTCRMN------PVELVR 112
Clustal Consensus             .                   *    :  :  :.  *    *                  11

                                   190       200       210       220       230       240
                            ....|....|....|....|....|....|....|....|....|....|....|....|
Agrobacterium sp. H13-3     LNTIANSESRTRRPTLAELDKIMEYFTKRNQATP--HVSRMDRVVAFAIYSTRRQEEIVR 222
Bradyrhizobium sp. BTAi1    LGITSKSRTRERRPSLAELDLLMQHFGERQQRRP--SSVPMQKVIAFAIFSTRRLEEITR 223
P. denitrificans PD1222     LGLVGKGNERDRRPSEEEIDAILKALESN---PR--QFIPVARVVRFAIATAMRQDEISR 214
B. gladioli BSR3            LGLTGKSVARDRRPTPDELNRILEYYTEMAKRER--AELPMRELIVFALFSTRRQEEITT 221
N. gonorrhoeae FA 1090      SNMVAKSAIRDRLPTTEELQTLTTYFLRQWQSRK--SSIPMHLIMWLAIYTSRRQDEICR 232
V. paradoxus S110           LGLVGKGRERDRRPTLDELQALADYFASN---SR--QIIPLGRIMRFAVATAMRQDEICR 215
A. baumannii D1279779       TRQISSSKVRDRLPTNEELVTLTKFFAERWKLNKYGTKYPMHLVIWFAIFSCRREAELTR 235
P. protegens Pf-5           MGAVSRSRERNRRPTLDELDRILTYFEQMRDRRR--QEIDMLRVIVFALFSTRRQEEITR 227
S. baltica OS155            QDLIAKSGKRSRRPTTDEIERLKVGLAARADQRA--AHIPYIDLLDFSILSCMRIGEVCR 227
S. enterica plasmid R64     --KPKVSSGRDRRLTSSEERRLSRYFREKNLM--------LYVIFHLALETAMRQGEILA 161
S. sonnei plasmid ColIb-P9  --KPKVSSGRDRRLTSSEERRLSRYFREKNLM--------LYVIFHLALETAMRQGEILA 161
E. coli ED1a                --KPKISSGRDRRLTSGEERRLSRYFRDKNQQ--------LYVIFHLALETAMRQGEILT 162
Clustal Consensus                 .  * *  :  *   :                     :. ::: :  *  *:   24
```

```
                                         250       260       270       280       290       300
                                ....|....|....|....|....|....|....|....|....|....|....|....|
Agrobacterium sp. H13-3         IEWEDLDEVHSRILVRDLKHPGQKKGNDVWCEIPP----EAMQIIKAMPKN--------- 269
Bradyrhizobium sp. BTAi1        LRRADLDEVGSKILVRDMKNPGEKLGNDVWCDLPA----EALHVAQSMPSD--------- 270
P. denitrificans PD1222         ARWEDYNPRTKMLLIRDRKDPRRKTGNDQRIPLLSVSGFDPCALIEEQRAL--------- 265
B. gladioli BSR3                IRVEDFE--GDRVLVRDMKHPGQKKGNDTWCDVPP----EAARVIEAVRPK--------- 266
N. gonorrhoeae FA 1090          LLFDDWHKNDCTRPVRDLKNPNGSTGNNKEFDILP----MALPVIDELPEESVRKRMLAN 288
V. paradoxus S110               IRWEDIDAKTRTVIVRDRKDPRDKNGNDQKVPLLDVTGFDAWAILEEQKPF--------- 266
A. baumannii D1279779           LWLQDYDSYHSSWKVHDLKNPNGSKGNHKSFEVLE----PCKTIVELLLDNEVRSRMLQL 291
P. protegens Pf-5               IRWDLLNESEQSALVTDMKNPGQKYGNDVWCHMPD----EAWRVLQSMPKV--------- 274
S. baltica OS155                ITWDDVDEAQKAVIVRDRKDPRKKAGNHMLVPMLG----GAWEILQKQP----------- 272
S. enterica plasmid R64         LRWEHIDLRHGVAHL-----PETKNGHSRDVPLSR----RARNFLQMMPVN--------- 203
S. sonnei plasmid ColIb-P9      LRWEHIDLRHGVAHL-----PETKNGHSRDVPLSR----RARNFLQMMPVN--------- 203
E. coli ED1a                    LRWEHLDLQHGVAHL-----PETKNGLPRDVPLSR----KARNYLQILPQQ--------- 204
Clustal Consensus                           .       :     *  . *       :            .          28

                                         310       320       330       340       350       360
                                ....|....|....|....|....|....|....|....|....|....|....|....|
Agrobacterium sp. H13-3         ---GPRIFPYGTAGVGAAFTRACQFLEIEDLHFHDLRHEGISRLFEMG-RTIPLAASVSG 325
Bradyrhizobium sp. BTAi1        ---TDEIFPYTTDAIGMGFTRACQLLGIVDLHFHDLRHDGVSRLFEMG-HNVPQVAAVSG 326
P. denitrificans PD1222         VGSRGRIFPYNGRSIGTAFRRVCRTLGIEDLHFHDLRHEATSRLFEAG-FTIEQVALVTG 324
B. gladioli BSR3                ---SGPIFPYNHRSISASFTKACAFLSIDDLHFHDLRHEGASRLFEMG-LNIPHVAAVTG 322
N. gonorrhoeae FA 1090          KGIADSLVPCNGKSVSAAWTRACKVLGIKDLRFHDLRHEAATRMAEDG-FTIPQMQRVTL 347
V. paradoxus S110               SGNSSLVFPYNGRSVGTAFRRACKELKIKDLKFHDLRHEAASRLFEAG-FTIEQAALVTG 325
A. baumannii D1279779           GYDERLLLPLNPKSIGKEFRDACKMLGIEDLRFHDLRHEGCTRLAEQS-FTIPEIQKVSL 350
P. protegens Pf-5               ---ADEVFPYNSRSVSASFTRACNFLEIEDLHFHDLRHDGVSRLFEMG-WDIPKVASVSG 330
S. baltica OS155                -RNDARVFPYNERSVTAGFQRVRNELGIEDLRYHDLRREGASRLFEKG-YSIDEVAQVTG 330
S. enterica plasmid R64         --LHGNVFDYTASGFKNAWRIATQRLRIEDLHFHDLRHEAISRFFELGSLNVMEIAAISG 261
S. sonnei plasmid ColIb-P9      --LHGNVFDYTASGFKNAWRIATQRLRIEDLHFHDLRHEAISRFFELGSLNVMEIAAISG 261
E. coli ED1a                    --INGNVFSYTSSGFKSAWRTALLDLKIENLHFHDLRHEAISRFFELGTLNVMEVAAISG 262
Clustal Consensus                     :.     ..   :  .    *  *  :*::****::.  :*:  *  .    :     ::   49

                                         370       380       390       400       410       420
                                ....|....|....|....|....|....|....|....|....|....|....|....|
Agrobacterium sp. H13-3         HRTWNSL-KRYTQIRERGDKFEGWKWLKTVTD--------------E------------- 357
Bradyrhizobium sp. BTAi1        HRSWSSL-KRYTHLRQTGNKYEGWKWLEVVTV--------------KTAPG--------- 362
P. denitrificans PD1222         HKDWKML-RRYTHIRPEGLHALAASRVA-------------------------------- 351
B. gladioli BSR3                HRSWSSL-KRYTHLRHVGDRWARWAWLDRVAP--------------LQEQS--------- 358
N. gonorrhoeae FA 1090          HDGWNSL-QRYVSVRKRSTRLDFKEAMMQAQS---------------DIKSGK------- 384
V. paradoxus S110               HKDWKML-KRYTHLRPEHLRGVRPKHIPPSLD----------------LPTGPTPADIAL 368
A. baumannii D1279779           HDSWSSL-QRYVSVKSRRNVIQLEEVLRLIDE---------------T------------ 382
P. protegens Pf-5               HRDWNSM-RRYTHLRGNGDPYAGWQWIERVIS--------------GPVIEAQVRVK--- 372
S. baltica OS155                HRNINTLWQVYTELFPKRLHDKFKD----------------------------------- 355
S. enterica plasmid R64         HRSMNML-KRYTHLRAWQLVSKLDARRRQTQKVAAWFVPYPAHITTIDEENGQKAHRIEI 320
S. sonnei plasmid ColIb-P9      HRSMNML-KRYTHLRAWQLVSKLDARRRQTQKVAAWFVPYPAHITTINEENGQKAHRIEI 320
E. coli ED1a                    HRSLNML-KRYTHLRAYQLVSKLDTKRKQTCKIAPYFVPYPATVGNRNGL-----FIVTL 316
Clustal Consensus               *    . : : *. :                                              54

                                         430       440       450       460       470       480
                                ....|....|....|....|....|....|....|....|....|....|....|....|
Agrobacterium sp. H13-3         ------------------------------------------------------------ 357
Bradyrhizobium sp. BTAi1        ------------------------------------------------------------ 362
P. denitrificans PD1222         ------------------------------------------------------------ 351
B. gladioli BSR3                ------------------------------------------------------------ 358
N. gonorrhoeae FA 1090          ------------------------------------------------------------ 384
V. paradoxus S110               GAWNNPERRAPQPNSAHP---------------------SQVAKPHHEHEALGLSASI-- 405
A. baumannii D1279779           ------------------------------------------------------------ 382
P. protegens Pf-5               ---RRAAGRAP------------------------------------------------- 380
S. baltica OS155                ------------------------------------------------------------ 355
S. enterica plasmid R64         GDFDNLHVTATTKEEAVHRASEVLLRTLAIAAQKGERVPSPGALPVNDPDYIMICPLNPG 380
S. sonnei plasmid ColIb-P9      GDFDNLHVTATTKEEAVHRASEVLLRTLAIAAQKGERVPSPGALPVNDPDYIMICPLNPG 380
E. coli ED1a                    HDFD-LETRAETRELAISHASVLLLRTLAQAAQRGERVPTPGELPANIDARVMICPLTS- 374
Clustal Consensus                                                                            54
```

	....	
***Agrobacterium* sp. H13-3**	----	357
***Bradyrhizobium* sp. BTAi1**	----	362
***P. denitrificans* PD1222**	----	351
***B. gladioli* BSR3**	----	358
***N. gonorrhoeae* FA 1090**	----	384
***V. paradoxus* S110**	----	405
***A. baumannii* D1279779**	----	382
***P. protegens* Pf-5**	----	380
***S. baltica* OS155**	----	355
***S. enterica* plasmid R64**	STPL	384
***S. sonnei* plasmid ColIb-P9**	STPL	384
***E. coli* ED1a**	----	374
**Clustal Consensus**		54
