# Supplementary figures and images for: A novel family of integrases associated with prophages and genomic islands integrated within the tRNA-dihydrouridine synthase A (dusA) gene
Source: Nucleic Acids Res. 2015 Apr 16;43(9):4547–57. doi: 10.1093/nar/gkv337 (PMC4482086; doi:10.1093/nar/gkv337)

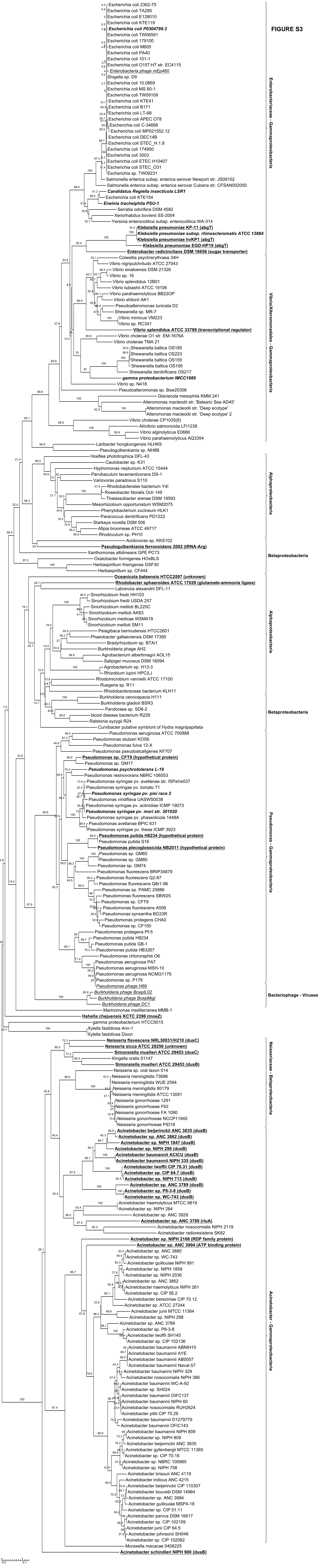

Supplement: SUPPLEMENTARY DATA [file supp_gkv337_nar-02196-h-2014-File012.pdf]
