# Supplementary material for: A novel family of integrases associated with prophages and genomic islands integrated within the tRNA-dihydrouridine synthase A (dusA) gene
Source: Nucleic Acids Res. 2015 Apr 16;43(9):4547–57. doi: 10.1093/nar/gkv337 (PMC4482086; doi:10.1093/nar/gkv337)
Supplement: SUPPLEMENTARY DATA [file supp_gkv337_nar-02196-h-2014-File013.pdf]

FIGURE S4

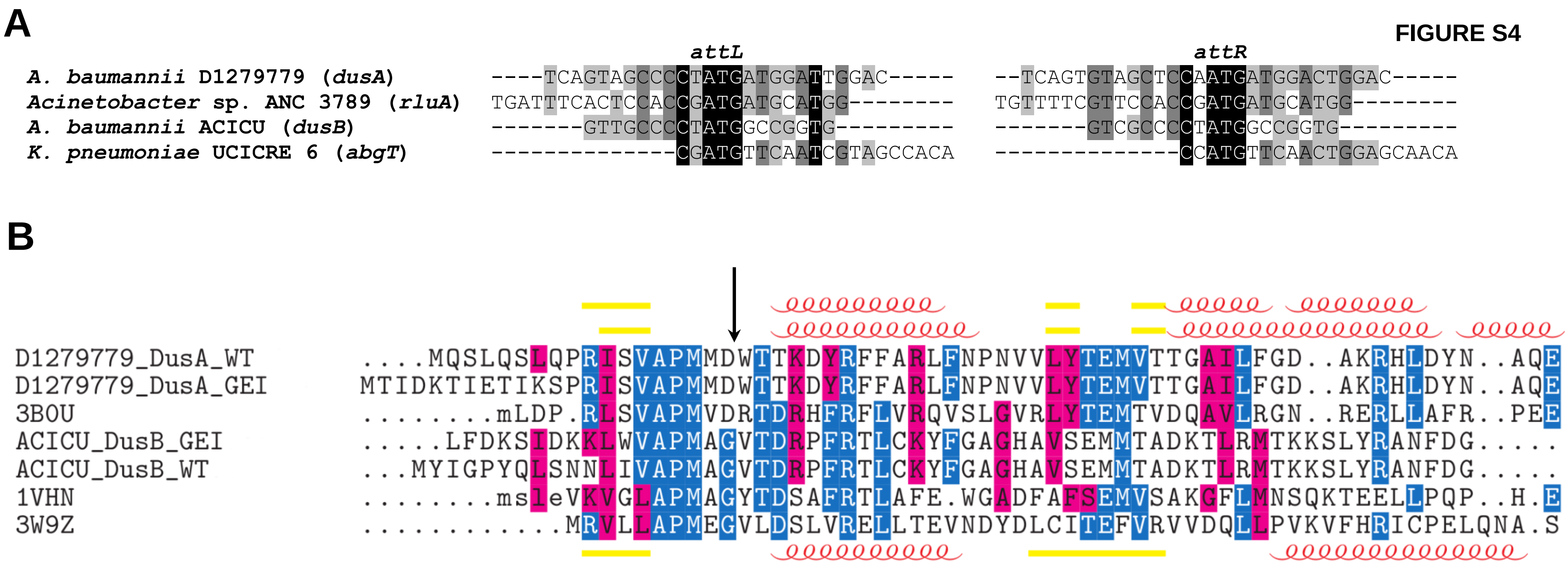
A
attL
attR
A. baumannii D1279779 (dusA) ----TCAGTAGCCCCTATGATGGATTGGAC----- --TCAGTGTAGCTCCAATGATGGACTGGAC-----
Acinetobacter sp. ANC 3789 (rluA) TGATTTCACTCCACCGATGATGCATGG-------- TGTTTTCGTTCCACCGATGATGCATGG--------
A. baumannii ACICU (dusB) -------GTTGCCCCTATGGCCGGTG--------- -------GTCGCCCCTATGGCCGGTG---------
K. pneumoniae UCICRE 6 (abgT) --------------CGATGTTCAATCGTAGCCACA --------------CCATGTTCAACTGGAGCAACA
B
D1279779_DusA_WT ....MQSLQSLQPRISVAPMMDWTTKDYRFFARLFNPNVVLYTEMVTTGAILFGD..AKRHLDYN..AQE
D1279779_DusA_GEI MTIDKTIETIKSPRISVAPMMDWTTKDYRFFARLFNPNVVLYTEMVTTGAILFGD..AKRHLDYN..AQE
3B0U ....mLDP.RLSVAPMVDRTDRHFRFLVRQVSLGVRLYTEMTVDQAVLRGN..RERLLAFR..PEE
ACICU_DusB_GEI .....LFDKSIDKKLWVAPMAGVTDRPFRTLCKYFGAGHAVSEMMTADKTLRMTKKSLYRANFDG.....
ACICU_DusB_WT ...MYIGPYQLSNNLIVAPMAGVTDRPFRTLCKYFGAGHAVSEMMTADKTLRMTKKSLYRANFDG.....
1VHN ....msleVKVGLAPMAGYTDSAFRTLAFE.WGADFAFSEMVSAKGFLMNSQKTEELLPQP..H.E
3W9Z ............MRVLLAPMEGVLDSLVRELLTEVNDYDLCITEFVRVVDQLLPVKVFHRICPELQNA.S

C

Acinetobacter baumannii D1279779

Pre-integration

TATTTAGCATGATTGATGCTTTCTTACAAAAAAAGCAAATGGTTTAGCTAAAGGTAATTTGTTAAAAATCATCTTTCTATATTTAACATAAGGGCGG
ABD1_05700 (MFS) stop

TTATAAATTGCGCAGTTAAATGCCACTGTCGATTCAGGGCTAAACCCCGTATAATGCTGCCCTTTCTTTAATTTAGCGACTTTTGGGTCTATCGGTT
-35 box -10 box

ATGCAATCACTTCAATCGTTACAACCTCGTATTTCAGTAGCCCCTATGATGGATTGGACAAACTAATAAAAATTATTATTTAAAAACAATGAATTAT
start *dusA* *attL*

Post-integration

CTTTTTGTTGATCCGGATAGCAGCTCGGTAACTAACGTTTCCATCTGCACCTTTTCTGGCAGTAATTGACCCCATAAATTTACACCATGAACCATTT
dusA-associated integrase start

TTTTCATTATGGTGTAAATTTTGGTGTAAATGCAAACCGATTAACGCAGCTAAACATGAGTTCCCACAGATTCAAACAGGTTGCAAAACACTGATTA
-35 box -10 box

AAGGTATGATTTTTA**ATGACTATTGATAAAACCATTGAAACTATTAAATCTCCAAGAATCAGTGTAGCTCCAATGATGGACTGGACTACAAAGGACT**
start *dusA* *attR*

Acinetobacter baumannii ACICU

Pre-integration

ATTTAAATAAAAAACGGGAAGACAACGGTTTGTTCATGGAGAGGAGGACACCTGCATAAAAAGATGAGCTGATCAAGCGGATGAAGTATACTATGCC
ACICU_02773 start -35 box -10 box

GATCATCAATTGTATGAATATAT**ATGTATATTGGTCCCTATCAACTGTCAAATAATTTAATTGTTGCCCCTATGGCCGGTGGGGTAGATATTTTATT**
start *dusB* *attL*

Post-integration

GTACTGTGCCGTTTTTAAGTTGACGTTTAGTGACTGTTCCCACGGAATTTGCACCACAAAATATTTAAGGTGCAAATAGGGTGCAATAAAAGGGCGC
dusB-associated integrase start

TAGAAGTCAATAAGAGGCGATTTAACGAAGTTAAACGAGGTTTGAAGAATTGAGCAAAAACACCGTTATAAAAGAATTGTTCGATAAATCAATAGAT
-35 box -10 box

AAGAAACTTTGGGTCGCCCCTATGGCCGGTG**TAACTGACCGTCCATTTAGAACGCTGTGCAAGTATTTCGGTGCGGGTCATGCAGTCAGTGAAATGA**
attR start *dusB*
