# Supplementary material for: A novel family of integrases associated with prophages and genomic islands integrated within the tRNA-dihydrouridine synthase A (dusA) gene
Source: Nucleic Acids Res. 2015 Apr 16;43(9):4547–57. doi: 10.1093/nar/gkv337 (PMC4482086; doi:10.1093/nar/gkv337)
Supplement: SUPPLEMENTARY DATA [file supp_gkv337_nar-02196-h-2014-File007.doc]

**Supplementary Data**

**Supplementary Table 1.** List of organisms encoding complete *dusA*-specific genomic islands, extracted from publicly available genome sequences. Tabulated features include accession version, published island names, genomic coordinates, size, G+C percentage and putative function.

**Supplementary Table 2.** Oligonucleotide sequences used in the detection of excised *dusA*-specific genomic islands and generation of markerless gene deletions. Relevant restriction sites are underlined.

**Supplementary Figure 1.** Multiple protein sequence alignment of representative tyrosine recombinase superfamily proteins used in the phylogenetic analysis outlined in Figure 1. The RHRH tetrad and nucleophilic tyrosine amino acid residues common to the tyrosine recombinase superfamily are highlighted black.

**Supplementary Figure 2.** Multiple protein sequence alignment of shufflon-specific DNA recombinases (Rci) against representative *dusA*-associated integrases (DAIs) outlined in Figure 1, with the addition of DAI sequences from *Shewanella baltica* OS155 (ABN63061), *Variovorax paradoxus* S110 (ACS18756), *Paracoccus denitrificans* PD1222 (ABL69522). Amino acid residues common to the representative DAIs are highlighted in dark blue, whilst conservatively substituted amino acid residues are highlighted dark green. Amino acids common (black), conservatively substituted amino acids (grey), and semi-conservatively substituted amino acids (.) in both Rci and DAI proteins are indicated. Previously mutagenised amino acid residues in R64 Rci (red) are also indicated.

**Supplementary Figure 3.** Phylogenetic analysis of 258 unique *dusA*-associated integrase proteins variants. Emboldened and italicised organism names are integrases that have an unknown target site, due to incomplete genome sequences. Emboldened and underlined organism names are integrases that are not associated with *dusA*, with their putative associated gene in brackets. Underlined and italicised organisms indicate integrases originating from bacteriophages. Unless otherwise indicated, all other integrase sequences are *dusA*-associated. The interior values are the bootstrap probabilities after 1000 replicates.

**Supplementary Figure 4.** **(A)** Alignment of predicted attachment sites from genomic islands associated with *dusA* and non-cognate sites in *rluA*, *dusB* and *abgT* genes. Nucleotides are shaded based on percentage identity: 50% (light grey), 75% (dark grey) and 100% (black).

(B) Alignment of N-terminal DusA and DusB protein sequences from *Acinetobacter baumannii* D1279779 and ACICU, prior to (D1279779_DusA_GEI, ACICU_DusB_GEI) and after excision (D1279779_DusA_WT, ACICU_DusB_WT) of its *dus*-specific genomic island, against crystallised dihydrouridine synthases from *Thermotoga maritima* MSB8 (1VHN), *Thermus thermophilus* HB8 (3B0U) and *Escherichia coli* K12 (3W9Z). Amino acid residues that are similar are highlighted pink, residues that are conserved are highlighted blue, and non-conserved residues are white. Amino acids in lower case are not part of the protein secondary structure. Yellow blocks and red spirals indicate beta strands and alpha helices, respectively. The two top secondary structure maps are that of 1VHN and 3B0U, whilst the bottom is that of 3W9Z. The arrow indicates the terminal amino acid in the genomic islands of *A. baumannii* D1279779 and *A. baumannii* ACICU, corresponding to the nucleotide sequences in Figure 4. This figure was generated with STRAP and has been truncated to emphasise the 5' end affected by island integration.

**(C):** Putative -10 and -35 promoters of the *A. baumannii* D1279779 *dusA* and ACICU *dusB* genes, prior to and after island integration. Protein coding genes sequences are emboldened, and putative attachment site sequences (*attL/attR*) are underlined and coloured red. Promoter analysis was conducted with BPROM.

References

1. Gyohda, A., Zhu, S., Furuya, N. and Komano, T. (2006) Asymmetry of shufflon-specific recombination sites in plasmid R64 inhibits recombination between direct sfx sequences. *J. Biol. Chem.*, **281**, 20772-20779.

2. Gyohda, A. and Komano, T. (2000) Purification and characterization of the R64 shufflon-specific recombinase. *J. Bacteriol.*, **182**, 2787-2792.

3. Gille, C. and Frömmel, C. (2001) STRAP: editor for STRuctural Alignments of Proteins. *Bioinformatics*, **17**, 377-378.
